# Environmental Gradients and Conservation Status Determine the Structure and Carbon‐Related Metabolic Potential of the Prokaryotic Communities of Mediterranean Inland Saline Shallow Lakes

**DOI:** 10.1002/ece3.71286

**Published:** 2025-05-26

**Authors:** Javier Miralles‐Lorenzo, Antonio Picazo, Carlos Rochera, Daniel Morant, Emilio O. Casamayor, Mateu Menéndez‐Serra, Antonio Camacho

**Affiliations:** ^1^ Cavanilles Institute of Biodiversity and Evolutionary Biology University of Valencia Paterna, Valencia Spain; ^2^ Centro de Estudios Avanzados de Blanes (CEAB‐CSIC) Girona Spain

**Keywords:** conservation status, co‐occurrence networks, inland saline shallow lakes, metabolic potential, microbial carbon‐related metabolisms, prokaryotic community structure

## Abstract

Mediterranean inland saline lakes may play an important role in the carbon cycle due to the metabolic capacities of their prokaryotic communities. However, these lakes are not homogeneous and have different environmental characteristics. In this work, the structure and both actual activity and predicted function of the prokaryotic communities inhabiting water and sediments of 15 Mediterranean inland saline shallow lakes have been studied. These lakes are grouped in categories according to their salinity, conservation statuses, and alterations, which determine the structuration of the prokaryotic communities and their carbon‐related metabolisms. Each salinity category and conservation status showed characteristic prokaryotic taxa. The relative abundance of methanogenic archaea tended to increase along the salinity gradient, but this did not result in increased methane emissions. The relationship between metabolic rates and the predicted abundance of their marker genes depended on the type of metabolism. Archaea played a relevant role in the organization of interactions between community members and were markers of good conservation status. Water communities were shaped by the salinity gradient, conservation status, and seasonality, while sediment communities were mostly determined by the salinity gradient. This work highlights the importance of combining molecular studies with in situ metabolic measurements to better understand carbon fluxes in inland saline aquatic ecosystems.

## Graphical abstract

Mediterranean inland saline shallow lakes are divided into different categories according to their salinity and conservation status, which determine the structuration of the prokaryotic communities and their carbon‐related metabolisms.

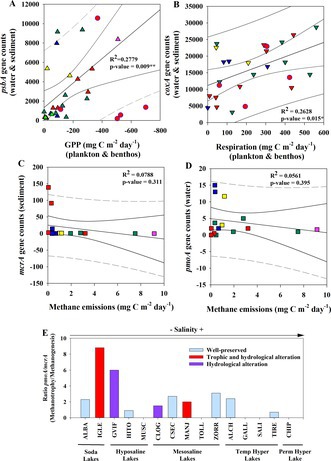

## 1 Introduction

Wetlands, including shallow lakes, play a crucial role in the carbon cycle, as well as in the nitrogen, phosphorus, and sulfur cycles (Ciais et al. 2014). These ecosystems are actively involved in the dynamics of carbon greenhouse gases (C‐GHG), such as carbon dioxide (CO_2_) and methane (CH_4_). The microbial communities that inhabit these kinds of ecosystems show metabolisms associated with atmospheric CO_2_ fixation and C‐GHG emissions, such as photosynthesis and both aerobic and anaerobic respiration, including methane production (Sica et al. 2016; Camacho et al. 2017; Morant et al. 2020a). The behavior of the different types of Mediterranean wetlands as net carbon sinks or GHG emitters mostly depends on the balance between autotrophic metabolisms, which fix carbon, and heterotrophic metabolisms, which generate GHG emissions. Different factors, such as salinity, seasonal variation, temperature, and the conservation status of wetland habitats, have a significant impact on the rates of these metabolic processes (Camacho et al. 2017; Morant et al. 2020a; Morant et al. 2020b). Information on the dynamics of these metabolisms in Mediterranean wetland ecosystems is limited, especially considering the regional diversity of wetland types, from inland freshwater lakes and ponds to inland saline lakes and coastal lagoons.

In limnological and microbial ecology studies, inland saline lakes have traditionally been poorly covered. However, over the last few years, they have reclaimed a prominent position. These lakes are predominantly found in arid and semi‐arid regions, where annual evaporation exceeds annual precipitation (Williams 2002). This determines that, when not altered, they are typically temporary systems with strong temporal fluctuations, drying up during the warmest months, and displaying important changes in water salinity along the year (Camacho et al. 2003; García‐Ferrer et al. 2003; Camuñas et al. 2018). The Iberian Peninsula harbors a large amount of inland saline lakes, almost unique in Western Europe, some of which are well‐preserved. Instead, others are affected by different anthropic alterations, like external freshwater inputs that, in some cases, include nutrient‐rich waters (Corrales‐González et al. 2019). These kinds of alterations lead to an artificial elongation of the hydroperiod and, whenever they are rich in nutrients, also cause eutrophication. These impacts can alter the structure and function of their prokaryotic communities.

Prokaryotic communities in wetlands play a crucial role in their functioning, as they are involved in the primary processes of the main biogeochemical cycles (Andreote et al. 2012). Consequently, the factors affecting these communities, particularly the groups of prokaryotes that participate in the different stages of the carbon cycle, have a significant impact on the carbon balance of inland saline shallow lakes and their eventual behavior as net carbon sinks or emitters. CO_2_ emissions from these wetlands are a balance between release and fixation. From a microbiological point of view, heterotrophic microorganisms produce the majority of CO_2_ released to the atmosphere. In the presence of oxygen, aerobic respiration predominates over the other types of respiration and is responsible for the remineralization of part of the organic matter; consequently, the role of this metabolism in the carbon cycle is extremely significant (Berg et al. 2022). Regarding CO_2_ fixation, this is primarily carried out by light‐dependent metabolisms that, among prokaryotes, can only be performed by specific groups, among which cyanobacteria stand out for their abundance and activity, being capable of oxygenic photosynthesis, although CO_2_ fixation can also be carried out to a lesser extent by other less relevant microbial metabolisms (Megonigal et al. 2004; Berg et al. 2010; Llirós et al. 2015).

Methane emissions depend on the balance between methane production by methanogenic archaea and methane consumption by aerobic prokaryotes, especially methanotrophic bacteria, but also by anaerobic prokaryotes, such as ANME archaea (Bridgham et al. 2013; Timmers et al. 2017). Methanogens (methanogenic archaea), which are strict anaerobes, function as terminal decomposers in oxygen‐depleted environments (Watanabe et al. 2009) and couple methane production, or methanogenesis, with energy production. There are three major methanogenic pathways (Evans et al. 2019): (1) methylotrophic methanogenesis, which may or may not rely on hydrogen and uses methylated substrates such as methylamines; (2) hydrogenotrophic methanogenesis, which is based on the reduction of CO_2_ by hydrogen; and (3) aceticlastic methanogenesis, which uses acetate. All three routes share an enzyme known as mcr (methyl‐coenzyme M reductase). This enzyme catalyzes the final step of methanogenesis, which is the release of methane. It is composed of multiple subunits, with the gene coding for the alpha subunit (*mcrA*) being widely used to study the diversity and transcriptional activity of methanogens (Watanabe et al. 2009; Wilkins et al. 2015; Evans et al. 2019). However, the relative importance of the three methanogenic pathways varies across ecosystems. Most methanogenic pathways are inhibited (outcompeted) by competition for metabolic substrates or electron donors between methanogenic archaea and sulfate‐reducing bacteria (SRB), which are better competitors than methanogenic archaea in high‐salinity environments due to the high concentration of sulfate required by SRB as electron acceptor. In saline ecosystems where SRB activity is high, methylotrophic methanogenesis predominates over other methanogenic pathways because it is the only process for which SRB do not compete with methanogens for substrates (Sorokin and McGenety 2019). Methanotrophic bacteria, on the other hand, can use the methane produced by methanogens as a carbon and energy source (Zheng et al. 2008). It has been reported that these microorganisms consume methane at high rates (Kolb et al. 2005), so their role in controlling methane emissions in saline shallow lakes may be significant.

Because of their temporality and short hydroperiod, Spanish saline lakes are very threatened by the forecasted higher temperatures and lower rainfall events due to global warming. There is little research available on the dynamics of the prokaryotic communities in saline lakes, despite being very abundant in the Iberian Peninsula, and even less specifically on the effect of climate change on the prokaryotic communities of these saline systems and their carbon‐related metabolisms, which strongly influence the carbon cycle in saline lakes and their global warming mitigation capacity. In fact, the molecular studies of the structuration and metabolic capacities of the prokaryotic communities in saline systems, especially when combined with in situ measurements of metabolic rates, are scarce worldwide and rely mostly on metabolic prediction bioinformatic pipelines with no comparison with the actual metabolic rates. Also, there is a lack of information on how the combination of the salinity gradient, conservation status, and seasonal variation shape the prokaryotic communities and their carbon‐related metabolisms in saline lakes.

Our study examines the factors that determine the structure and carbon‐related metabolisms of prokaryotic communities in the water and sediment of 15 inland saline shallow lakes within the Spanish Mediterranean region. Based on their salinity and conservation status, these lakes were separated into distinct groups. The predicted relevance of the different metabolisms involved in the C‐GHG emissions has been compared with the actual metabolic rates. We hypothesize that both salinity and the conservation status, in combination with seasonal changes, determine the structure and carbon‐related metabolic potential of the prokaryotic communities of the studied saline shallow lakes. Further, we also hypothesize that lake sediment microbiota are more resilient as seasonal environmental changes in sediments are less intense than in water. Our results show the significance of combining molecular techniques with in situ metabolic measurements for a comprehensive understanding of the role of prokaryotic communities in carbon greenhouse gas emissions (C‐GHG) in Mediterranean inland saline shallow lakes, aiming for a thorough understanding of the factors regulating carbon fluxes in these types of ecosystems.

## 2 Materials and Methods

### 2.1 Study Sites

Fifteen inland saline shallow lakes located in the Mediterranean biogeographic region of Spain were selected as representative of the different saline lake types and conservation status in Western Europe (Table 1). The studied lakes remain dry over the summer period and have a maximum depth always lower than 1 m, as much as that reached at the hydrological yearly optimum. These lakes were grouped into five categories according to their salinity: soda lakes, hyposaline, mesosaline, and hypersaline lakes, all of them being temporary lakes. The fifth category is represented by the only permanent hypersaline inland lake in Spain, Salada de Chiprana, which, due to historical alterations related to the development of agriculture, has been constantly flooded for several centuries (Valero‐Garcés et al. 2000), and nowadays is considered to be naturally permanent.

**TABLE 1:** Main characteristics of the studied inland saline shallow lakes.

Lake	Code	Salinity category	Conservation status	Hydroperiod
Laguna de Caballo Alba	ALBA	Soda lake	WP	Temporary
Laguna de la Iglesia	IGLE	Soda lake	HTA	Semi‐permanent
Laguna Grande de Villafranca	GVIF	Hyposaline	HA	Permanent
Laguna de El Hito	HITO	Hyposaline	WP	Temporary
Laguna de Musco	MUSC	Hyposaline	WP (restored)	Temporary
Laguna de Carralogroño	CLOG	Mesosaline	HA	Semi‐permanent
Laguna de Carravalseca	CSEC	Mesosaline	WP	Temporary
Laguna de Manjavacas	MANJ	Mesosaline	HTA	Semi‐permanent
Laguna de los Tollos	TOLL	Mesosaline	WP (restored)	Semi‐permanent
Laguna Salada de Zorrilla	ZORR	Mesosaline	WP	Temporary
Laguna de Alcahozo	ALCH	Hypersaline	WP	Temporary
Laguna de Gallocanta	GALL	Hypersaline	WP	Temporary
Laguna de Salicor	SALI	Hypersaline	HTA	Temporary
Laguna de Tírez	TIRE	Hypersaline	WP	Temporary
Laguna Salada de Chiprana	CHIP	Permanent Hypersaline	WP	Permanent

Abbreviations: HA, hydrological alteration; HTA, hydrological and trophic alteration; WP, well‐preserved.

The classification used considers conductivity as a proxy for salinity, following Hammer (1986). In our study, lakes with conductivities at the hydrological optimum below 15 mS·cm^−1^ were classified as hyposaline lakes, those with conductivities between 15 and 45 mS·cm^−1^ as mesosaline lakes, and the hypersaline lakes were those with conductivities always above 45 mS·cm^−1^. Laguna de Caballo Alba and Laguna de la Iglesia, due to their high sodium carbonate levels and permanently elevated pH, can typically be classified as soda lakes. Taking into account the nature and effects of human water inputs on the ecological characteristics of the lakes, they were classified as well‐preserved lakes, hydrologically altered lakes (Laguna de Carralogroño and Laguna Grande de Villafranca) and lakes where a hydrological alteration, in turn, led to an additional trophic alteration (Laguna de Manjavacas, Laguna de la Iglesia and Laguna de Salicor) (Morant et al. 2024). The well‐preserved lakes were those lacking a strong hydrological alteration, thus maintaining their natural hydroperiod, and at the same time did not present significant trophic alterations. Among the well‐preserved lakes, two of them, Laguna de Musco and Laguna de los Tollos, had been restored and had recovered their natural characteristics, therefore being considered as equivalents of well‐preserved lakes. Lakes with hydrological alteration received significant extra freshwater inputs but with a low nutrient load. These artificially increased water inputs led to longer hydroperiods, becoming permanent or semi‐permanent lakes, and also to lower salinity, but did not increase the trophic status of the lakes. Lakes with both hydrological and trophic alteration were those receiving water inputs, sometimes from wastewater treatment plants, which altered their hydroperiod and increased their nutrient availability, leading to average chl‐*a* concentrations above 15 mg m^3^.

### 2.2 Sampling and Physical and Chemical Analysis

In the sampling campaigns, water and sediment samples were taken at the same central point in each lake. These campaigns were carried out three times: the first one during the filling period (fall–winter), another in the ecological optimum (spring), and finally, during the drying phase (summer) of the 2016–2017 hydrological cycle. The water samples were collected at a depth of 20 cm in sterile containers that were kept cold until they reached the laboratory. For all samples, between 200 and 400 mL of the water were filtered through 0.22 μm pore size polycarbonate filters (Nucleopore, Whatman) to obtain total DNA and were kept at −20°C until DNA extraction. For dissolved nutrient analyses, the water samples were filtered through glass fiber filters (Whatman GF/F), and the filtered water was kept frozen until further analysis. Sediment samples were collected in triplicate in sterile containers and kept cold until processing in the laboratory within 24 h at maximum, where the sediments were homogenized by mixing with a metal rod. Samples for DNA extraction were placed in 1.5 mL Eppendorf tubes and kept at −20°C until extraction. For DNA extraction, about 300 mg of sediment per sample were used.

For the water samples, dissolved oxygen, temperature, conductivity, and pH were measured in situ at the depth of water sampling. Dissolved oxygen and temperature were measured using a WTW Multi 3140 multiparameter probe with an FDO 925 sensor, with a salinity correction (if necessary) applied to the oxygen measurements. The maximum depth was obtained using a limnimeter located at the deepest point of the lakes. Conductivity was measured using a WTW Tetracon 925 IDS conductivity meter, and pH was measured using a Crison pH Basic 20 pH meter. Analyses of dissolved inorganic nutrients (nitrogen and phosphorus) and other environmental water variables were mostly performed following the standard methods described in APHA (2005). Briefly, the nitrate concentration was determined using the second derivative method by UV/visible spectroscopy (APHA 4500 ${\mathrm{NO}}_{3}^{-}$ C). Ammonium concentration was obtained by the modified method based on the indophenol blue method (Golterman 2007). Soluble reactive orthophosphate (SRP) was determined by the method based on the ascorbic acid and phosphomolybdic acid method (APHA 4500 P E). Sulfate concentration was obtained by precipitation as barium sulfate (APHA 4500 ${\mathrm{SO}}_{4}^{2-}$ E). Total suspended solids (TSS) were determined by drying at 105°C (APHA 2540 B Solids). Particulate organic matter (OM) was determined by the Loss‐on Ignition (LoI) at 460°C for 6 h (Heiri et al. 2001). Dissolved organic carbon (DOC) was measured using a TOC‐VCHS Shimadzu analyzer (Tokyo, Japan). Alkalinity was measured by titration with hydrochloric acid using phenolphthalein as the pH indicator. Chlorophyll‐*a* (Chl‐*a*) was quantified by filtering the water samples with glass fiber filters (Whatman GF/F), which were immersed in acetone at −20°C to extract the photosynthetic pigments. Once extracted, the Chl‐*a* concentration was obtained following Picazo et al. (2013).

For the sediment analyses, the different measurements were carried out in triplicate. The sediment was sampled at surface level, at an approximate depth of 5 cm. The pH and conductivity were measured by 1/5 dilution with distilled water (Rayment and Higginson 1992). Conductivity was obtained using a WTW LF‐191 conductivity meter, and pH using a Crison pH Basic 20 pH meter. In addition, the percentages of organic matter and carbonates were determined respectively by the LoI at 460°C for 6 h, and at a temperature of 950°C for 4 h (Heiri et al. 2001).

Gross primary production (GPP) and aerobic respiration rates were measured for both plankton and benthos of the studied lakes by in situ measurements of the variation in oxygen concentration in incubations in light and dark, which were transformed into carbon, assuming that 1 mole of O_2_ generated or consumed implies 1 mole of CO_2_ consumed or generated (Morant et al. 2020b; Morant 2022). Methane emissions, which are a balance between microbial production and consumption of this gas, were determined using the procedure described in Camacho et al. (2017), obtaining sediment cores for each lake and sampling time using transparent methacrylate tubes that were incubated in climatic chambers at a temperature corresponding to that of the sampling period, for 1–4 days depending on the lake. Methane generated during that time was measured by gas chromatography or by an Aeroqual Gas AQ‐MT sensor equipped with a gas‐sensitive semiconductor (Aeroqual, New Zealand) calibrated by gas chromatography (Camacho et al. 2017; Morant et al. 2020b; Morant 2022). Methane concentration measurements were transformed into carbon in the form of methane (C–CH_4_) emission rates per unit time (Morant et al. 2020b; Morant 2022).

### 2.3 DNA Extraction, Sequencing, and Taxonomic Assignment

DNA extraction from water filters and sediment samples was carried out using the EZNA soil DNA isolation kit (Omega Bio‐Tek Inc., Norcorss, GA, United States) following the instructions of the supplier. The sequencing of the V4 region of the 16S rRNA gene was performed with the Illumina MiSeq System (2 × 250 bp), following the procedure described by Picazo et al. (2019). For each sample, Illumina compatible and dual indexed amplicon libraries of the V4 hypervariable region of the 16S rRNA gene were obtained with the primers 515f (GTGCCAGCMGCCGCGGTAA) and 806r (GGACTACHVGGGTWTCTAAT) (Kozich et al. 2013). Then, completed libraries were batch normalized using Invitrogen SequalPrep DNA Normalization Plates. After, the product recovered from the plates was pooled and quantified using a combination of Qubit dsDNA HS, Agilent 4200 TapeStation High Sensitivity DNA, and Kapa Illumina Library Quantification qPCR assays. The pool obtained was loaded on a standard Illumina MiSeq v2 flow cell and sequencing was carried out in a 2 × 250 bp paired end format using a MiSeq v2 500 cycle reagent cartridge. Custom sequencing and index primers complementary to the 515/806 target sequences were added to appropriate wells of the reagent cartridge. Base calling was carried out by Illumina Real Time Analysis (RTA) v1.18.54 and the output of RTA was demultiplexed and converted to FastQ format with Illumina Bcl2fastq v2.19.1.

All the sequence data from this study have been deposited in the NCBI Sequence Read Archive under BioProject ID PRJNA819854. The sequences were processed using the UPARSE pipeline (Edgar 2013). After the merging of R1 and R2 read pairs, the sequences were filtered by a maximum expected error of 0.5, and chimeric reads were removed by the UCHIME algorithm. Filtered sequences were clustered in Zero‐radius Operational Taxonomic Units (ZOTUs), which are sequences at 100% identity. Taxonomic assignment was carried out with SINA aligner v.1.2.11, using the SILVA 138.1 reference database. ZOTUs with low alignment scores (< 90%) were filtered, and sequences classified as mitochondria or chloroplasts were removed. The resulting ZOTU table consisted of 10,755 ZOTUs for 78 samples. Rarefactions were performed separately for the water and sediment samples. To avoid the loss of less abundant ZOTUs, rarefactions were repeated 100 times (Caliz et al. 2015) and then unified into two different average ZOTU tables with thresholds of 5783 reads/sample for water and 3950 reads/sample for sediment. Two samples were removed as they did not reach the minimum threshold. In order to increase the number of minoritarian reads, it was decided to take singletons (sequences with a single read) into account and to change the reference database from SILVA to RDP as this currently has a better representation of the Archaea domain. Using the RDP classifier 2.13 tool (Wang et al. 2007), a taxonomic assignment was performed for all reads (trimmed and filtered by quality by trimmomatic) from raw FastQ R1‐forward files using the RDP database version 11.5 as a reference. Afterwards, only those reads with low alignment quality (standard RDP cutoff 80%) were filtered out, obtaining a sequence table that was rarefied at 5200 reads/sample. This rarefied table was used specifically for the analysis of the Archaea domain in sediment.

### 2.4 Metabolic Potential of the Prokaryotic Communities

The bioinformatic tool PICRUSt2 (phylogenetic investigation of communities by reconstruction of unobserved states) (Douglas et al. 2020) was used to describe the potential metabolisms that could be performed by the prokaryotic aquatic and sediment communities. In order to do this, the raw ZOTU table prior to the filtering and rarefaction was used as PICRUSt2 has its own normalization procedure. A selection of the inferred genes that participate in the carbon‐related metabolisms measured in the field was performed. The inferred genes for carbon‐related metabolisms were the *psbA* gene for photosynthesis (Sander et al. 2010), the *coxA* gene for aerobic respiration (Kessler et al. 2019), the *pmoA* gene for aerobic methanotrophy (Chen et al. 2020) and the *mcrA* gene for methanogenesis (Kessler et al. 2019; Chen et al. 2020). For sulfur‐related metabolism, the selected gene marker was *dsrB* for dissimilatory sulfate reduction (Roy et al. 2018). The presence or absence of each of these marker genes in each of the ZOTUs was also determined with PICRUSt2. In addition, the different methanogenic pathways were explored with the inferred genes *mtmB* and *mtbB* (methylotrophic methanogenesis), *ackA* (aceticlastic methanogenesis) and *mch* (methanogenesis from CO_2_) (Zhou et al. 2022).

### 2.5 Prokaryotic Co‐Occurrence Networks

For water and sediment, two different co‐occurrence networks were constructed based on the rarefied ZOTU tables through CoNet software (Faust et al. 2012; Faust and Raes 2016) with Silva 138.1 taxonomic assignment.

Each ZOTU was assigned to a specific level of the factors salinity category, conservation status, and season if its abundance in any of these levels was higher than 70% of its total reads. The different levels of the salinity category factor were the different salinity categories into which the lakes were grouped: soda lakes, hyposaline, mesosaline, temporary hypersaline, and permanent hypersaline. The conservation status factor was divided into well‐preserved, restored, hydrologically altered, and hydrologically and trophic altered lakes. The season factor was divided into fall–winter (filling), spring (ecological optimum) and summer (drying) periods. The ZOTUs that did not reach a minimum abundance of 70% of their total reads in any level of the factors were divided into two classes. On the one hand, if within the factors salinity category and conservation status, a ZOTU had reads at all levels of that factor, it was considered cosmopolitan. If the same was true for the season factor, with reads in all seasons, it was considered to be present throughout the year (Core). On the other hand, if a ZOTU had no reads at any of the levels of a factor, it was considered not cosmopolitan/not core (NC). Co‐occurrence and co‐exclusion relationships between network nodes were derived with different metrics. The minimum reads by each ZOTU taken into account to construct the networks were 25 reads for water and 50 reads for sediment. The metrics used were Pearson's correlation, Spearman's correlation, Bray–Curtis dissimilarity, and Kullback–Leibler dissimilarity. Environmental data were included in the analysis. Up to 1000 upper and lower edges were considered. Edge significance was assessed using a combination of permutations and bootstraps generated with 100 iterations and with a renormalization routine to avoid compositionality bias and thus mitigate the establishment of spurious correlations between nodes. The final edge significance was obtained by fusing the *p*‐value of the edges of each metric with Brown's method and with a multiple testing correction using the Benjamini‐Hochberg method. The final network was visualized using the network visualization software Cytoscape (Lopes et al. 2010). In these general networks, the clustering coefficient, which gives a measure of the complexity of the network (Guo et al. 2022), was determined, and also the modularity (with the MCL algorithm), which reports the degree to which a network is divided into different subnetworks or modules, which are made up of members that have more relationships with each other than with the members of the other modules (Khan et al. 2019). Also, in these networks, the total degree of the most abundant families was defined as the sum of the degree (number of connections) of the individual nodes that were assigned to these families. The total abundance of each family was calculated by summing the abundance of the individual nodes that were assigned to each family.

The topological roles of the individual nodes that formed the networks were calculated according to the simplified classification of Olesen et al. (2007), which divides them into four types: peripheral nodes, which maintain few connections with the rest of the nodes; connector nodes, which connect modules of the network and are very important for maintaining the network; nodes called module hubs, which are very important for maintaining the organization of the module of which they are part; and nodes called network hubs, which are important for maintaining the coherence of both the module where they are located and the overall network. The membership of a node in one of these particular groups was established by calculating its within‐module connectivity (*Z*_*i*_) and among‐module connectivity (*P*_*i*_) (Olesen et al. 2007), and the result was visualized by means of a *Z*_*i*_ – *P*_*i*_ graph, where the nodes are placed in four regions, corresponding to each of the four possible roles, based on their *Z*_*i*_ and *P*_*i*_ values. For this analysis, the water and sediment networks were reconstructed, but taking into account only the co‐occurrence relationships and not the co‐exclusion relationships. The same metrics and minimum values of ZOTU reads as before were used.

### 2.6 Statistical Analyses

Statistical analyses were carried out with Primer 7 and R software. Principal Coordinates Analysis (PCOs) was performed with the Euclidean distance matrix obtained from the normalized environmental variables. The water and sediment rarefied ZOTU tables were used to obtain the discriminant prokaryotic biomarkers for the different salinity categories and conservation statuses through the LEfSe algorithm (Segata et al. 2011), and to perform the variation partitioning analysis. The same ZOTU tables for water and sediment were standardized and square root transformed prior to obtaining their Bray–Curtis similarity matrices. These matrices were used to test for statistical differences between the prokaryotic communities of the different salinity categories and conservation statuses using a PERMANOVA analysis (999 permutations) (Anderson 2001). Also, these matrices were used to perform BVStep analyses (Clarke and Warwick 1998) at the family level to obtain the subset of families that best characterize the general patterns of the community structure of the water and sediment. Significant families from BVStep and LEfSe analyses were used to perform a distance‐based Redundancy Analysis (dbRDA) (Legendre and Anderson 1999) to observe the effect of the environmental variables on the ordination of the prokaryotic communities. For water, the matrix of normalized environmental variables for the dbRDA was made up of maximum depth, water oxygen, temperature, conductivity, pH, chlorophyll‐*a*, suspended organic matter, dissolved organic carbon (DOC), alkalinity, soluble reactive orthophosphate, nitrate, ammonia, and sulfate. For sediment, the matrix of normalized variables consisted of conductivity, pH, sediment organic matter, and carbonate. In these matrices, the gross primary production (GPP), the aerobic respiration, and methane emissions rates of the lakes were included. The data of the metabolic rates are those provided by Morant (2022).

## 3 Results

### 3.1 Environmental Characteristics of the Inland Saline Lakes

The values of the main environmental water variables of the studied lakes are shown in Table S1. These lakes showed a high salinity gradient. At the extreme, with lower conductivities, are the soda lakes and the hyposaline lakes, which presented relatively low values, such as 3.7 mS·cm^−1^ in Laguna de Caballo Alba, while the highest conductivities were observed in the temporary and permanent hypersaline lakes with conductivities greater than 50 mS·cm^−1^. The highest average pH and alkalinity values were found in the soda lakes, with pHs higher than 9 and alkalinities higher than 26 meq·L^−1^, while the rest of the lakes presented mean pH values around 8 and average alkalinities lower than 20 meq·L^−1^. Mean particulate water organic matter values increased along the salinity gradient, reaching the highest values in the temporary hypersaline lakes, and presenting high values in the lakes with hydrological and trophic alteration, where the mean chlorophyll‐*a* concentration also reached the highest levels.

**FIGURE 1:**
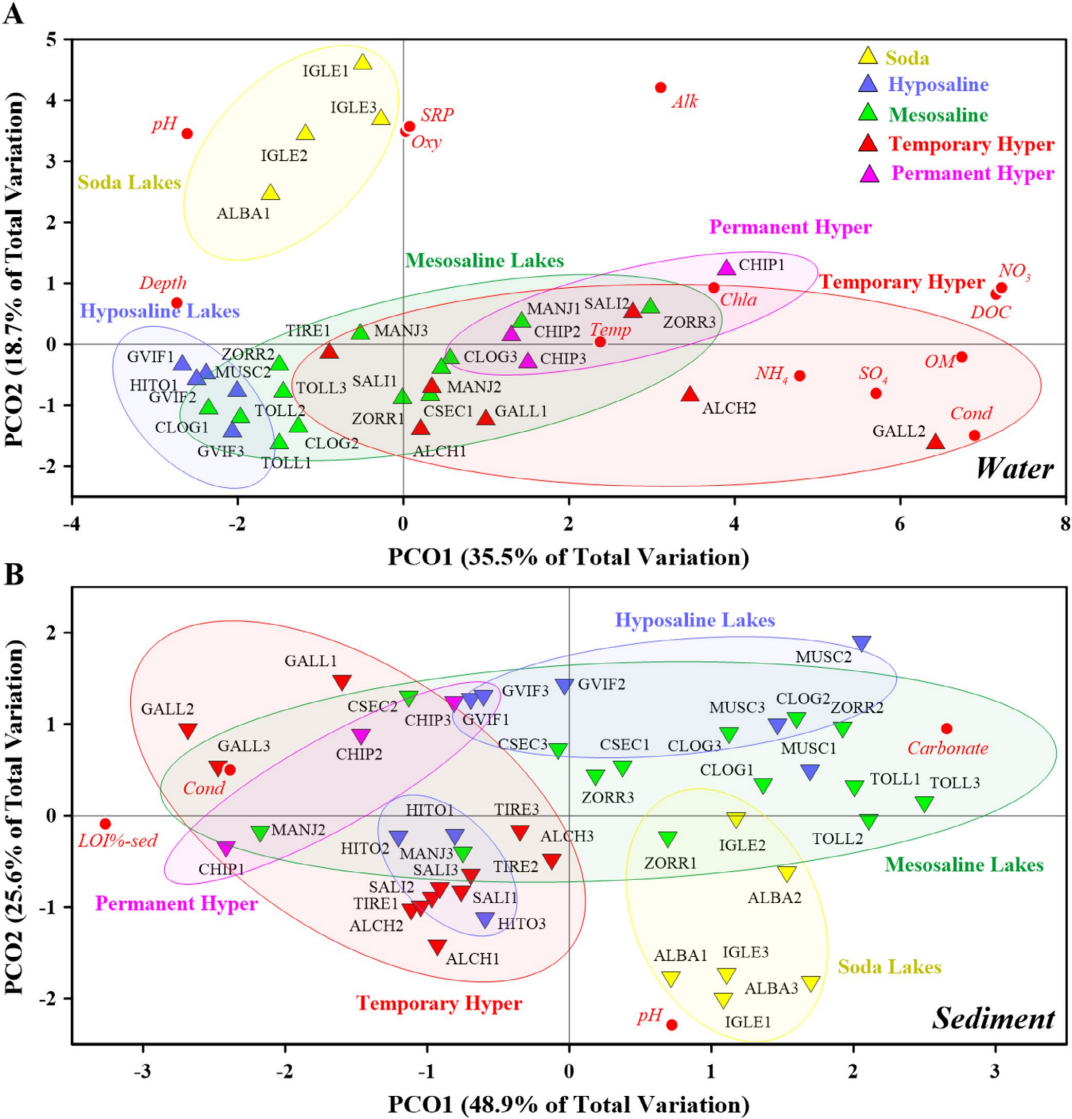
PCO of the water (A) and sediment (B) samples based on their environmental variables. The lake codes are described in Table 1. The numbers adjacent to the lake codes indicate the sampling periods: 1 (filling), 2 (ecological optimum) and 3 (drying). Water environmental variables: Chla (chlorophyll‐*a*), Alk (alkalinity), OM (suspended organic matter), DOC (dissolved organic carbon), NH4 (ammonium), Oxy (oxygen), Temp (temperature), SRP (soluble reactive orthophosphate), Cond (conductivity), NO_3_ (nitrate), SO_4_ (sulfate), depth (maximum lake depth), and pH. Sediment environmental variables: LOI%‐sed (sediment organic matter), cond (conductivity), pH, and carbonate.

Regarding the ordination of the water samples based on their environmental variables (Figure 1A), conductivity was a key environmental factor to separate the different lakes. The soda lakes were the most different, as they were separated from the others due to their high pH, alkalinity, SRP, and oxygen values. The rest of the salinity categories partially overlapped in the ordination, as generally, and following the salinity gradient upwards, the lakes with the highest conductivity values within a category had conductivity values lower but close to the lakes with the lowest conductivities in the next category. Despite this, each salinity category presented specific characteristics. Temporary and permanent hypersaline lakes showed high values of particulate organic matter, sulfate, and nitrate. Mesosaline lakes showed more moderate values of particulate organic matter, while hyposaline lakes showed the lowest values of particulate organic matter, sulfate, nitrate, and ammonium.

Regarding the ordination of the sediment samples (Figure 1B), these followed similar patterns to the water samples, with conductivity being the most relevant environmental variable to differentiate the different salinity categories. In turn, the soda lakes were the most different, while the other salinity categories were found to partially overlap, as mentioned for the water samples. However, these salinity categories also showed specific characteristics of their sediments. Thus, soda lakes showed high pH values, while hypersaline lakes showed high organic matter values, and the mesosaline lakes had higher carbonate values. On the other hand, hyposaline lakes did not form a well‐defined group but had in common low to moderate conductivity values.

### 3.2 Prokaryotic General Patterns and Characteristic Taxa

**FIGURE 2:**
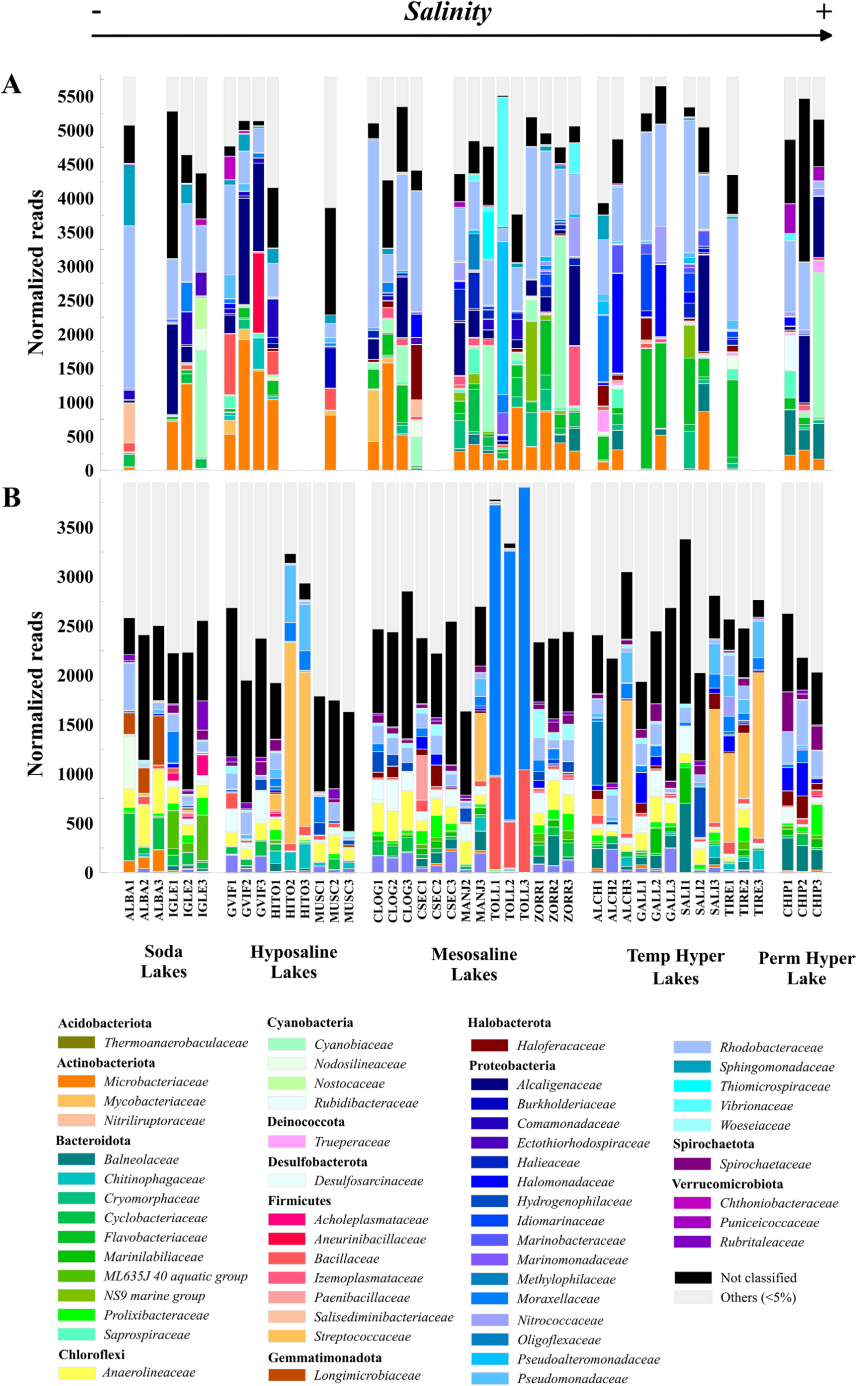
Relative abundances of prokaryotic families in the water (A) and sediment (B) of the studied lakes. The height of the overall bar in each sample corresponds to the rarefaction threshold (5783 reads/sample for water and 3950 reads/sample for sediment). Families are grouped by phylum. In the water, empty bars indicate that the lake was dry at that sampling period. In the legend, “Other” refers to families with a relative abundance of less than 5%.

Taxonomic assignation of the amplicons of the 16S rRNA gene resulted in 55 phyla for water and 66 for sediment. In general (Figure 2), the most represented phylum both in water and sediments was Proteobacteria, to which the family Rhodobacteraceae belongs. This family was distributed along the entire salinity gradient. With respect to water, another dominant phylum was Cyanobacteria, due specially to the families Cyanobiaceae and Nodosilineaceae, but in this case the abundance of Cyanobacteria increased in the warmer months, especially in the lakes that showed hydrological and trophic alteration. In the sediment, the phylum Firmicutes was highly represented in the well‐preserved lakes, especially in the temporary hypersaline lakes and in Laguna de El Hito, linked to the high relative abundance of the Streptococcaceae family, which is associated with fecal contamination from waterfowl. Regarding the statistical differences between the prokaryotic communities of the different salinity categories, both in the water and in the sediments, the communities of all the salinity categories were significantly different from each other (*p* < 0.01). Regarding the communities of lakes with contrasting conservation statuses, there were also significant differences between them (*p* < 0.01) both in the water and the sediments.

**FIGURE 3:**
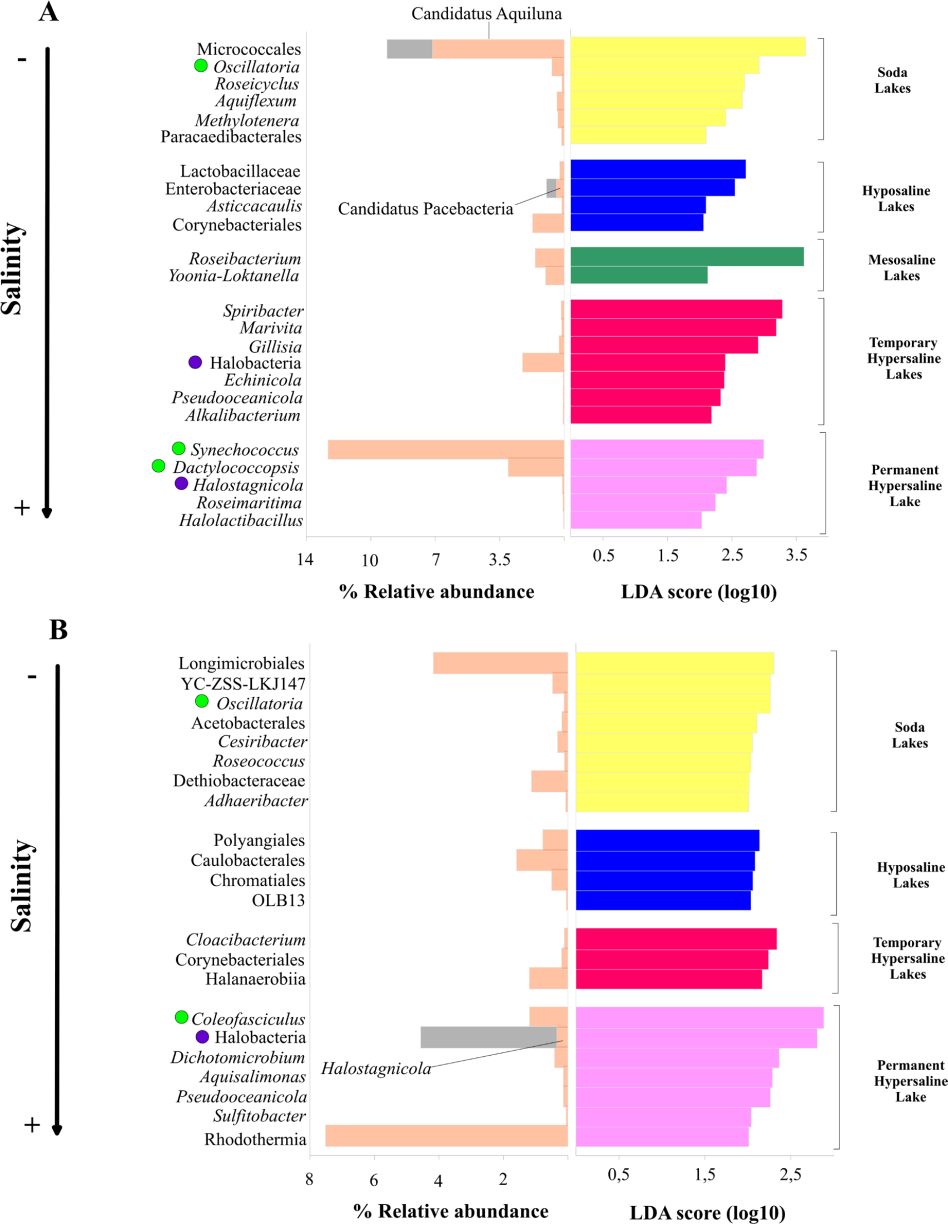
Characteristic taxa obtained with the LefSe discriminant analysis for the different salinity categories in which the lakes are grouped for water (A) and sediment (B). The LDA score and the average relative abundance of each taxon are shown. In the stacked bars, the abundance of the highest level taxon always corresponds to the largest bar while the abundance of the lowest level taxon, which is responsible for a part of the abundance of the higher level taxon, is represented by a small bar. Cyanobacterial taxa are highlighted with a green circle and Archaea with a purple circle.

**FIGURE 4:**
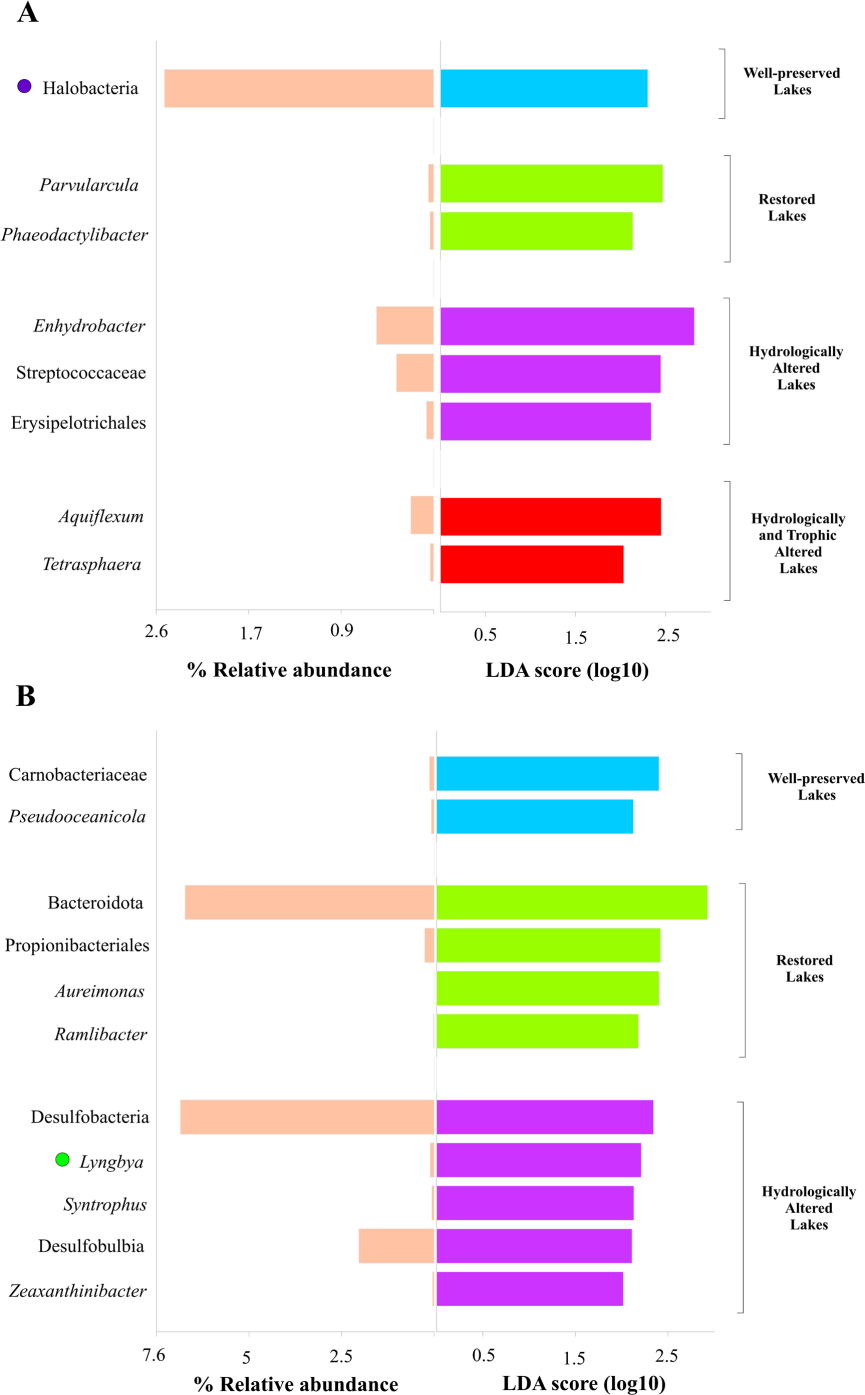
Characteristic taxa obtained with the LEfSe discriminant analysis for the different conservation statuses in which the lakes are grouped for water (A) and sediment (B). The LDA score and the average relative abundance of each taxon are shown. Cyanobacterial taxa are highlighted with a green circle and Archaea with a purple circle.

On the other hand, the results of the LefSe discriminant analysis showed that both in water and sediments the different salinity categories and conservation statuses in which the lakes are grouped presented characteristic taxa (Figures 3 and 4). Moreover, this specificity of taxa (indicated by the LDA score, the higher the value the stronger discriminant capacity of the taxon) was not directly related to the abundance of these taxa in the total prokaryotic community, as some had a high relative abundance while others displayed low abundance in the overall community. In water, the bacterial characteristic taxa of the different salinity categories and conservation statuses were distributed at all the levels of these factors, while the archaeal characteristic taxa were found only in the lakes with the highest salinity or a good conservation status, the latter of interest for its possible use as indicator taxa.

In the water (Figure 3A), the most characteristic taxon in the soda lakes was the genus *Candidatus* Aquiluna, which also showed a high relative abundance, while the next most differential taxa were phototrophic organisms, namely the cyanobacterial genus *Oscillatoria* and the phototrophic bacterial genus *Roseicyclus*, though both with a low relative abundance. In the hyposaline lakes, the most characteristic taxa were the family Lactobacillaceae as well as the genus *Candidatus* Pacebacteria within the family Enterobacteriaceae, while the mesosaline lakes showed few characteristic taxa, such as the genera *Roseibacterium* and *Yoonia‐Loktanella*. The taxa that characterized the temporary hypersaline lakes were generally halophilic, such as the genus *Spiribacter*. Furthermore, in temporary hypersaline lakes, archaea were relevant as characteristic microorganisms, since the archaeal class Halobacteria, formed by halophilic microorganisms, was the differential taxon with the highest relative abundance. As for the permanent hypersaline lake, cyanobacteria stood out as characteristic organisms, since two genera, *Synechococcus* and *Dactylococcopsis*, showed a high relative abundance in the Salada de Chiprana as well as a high weight as characteristic taxa for this permanent hypersaline lake, while the third taxon with the highest weight as a marker, the halophilic archaea *Halostagnicola*, had very low relative abundance.

With regard to the sediments (Figure 3B), the most characteristic taxon corresponding to the soda lakes was the order Longimicrobiales, with a high relative abundance. In the hyposaline lakes, the most characteristic taxa were made up of three orders (Polyangiales, Caulobacterales and Chromatiales), which showed moderate relative abundance. The mesosaline lakes, however, did not show any characteristic taxa. For the temporary hypersaline lakes, the taxon with the highest relative abundance was the bacterial class Halanaerobiia, while in the permanent hypersaline lake Salada de Chiprana, the most characteristic taxon was the cyanobacterial genus *Coleofasciculus*, which builds microbial mats in the shallower areas of this lake, although the halophilic archaea *Halostagnicola* was the second most characteristic taxon, and the bacterial class Rhodothermia, formed mostly by halophilic microorganisms, showed a high relative abundance.

In relation to the conservation status, in the water (Figure 4A), the characteristic taxon of the well‐preserved lakes was the archaeal class Halobacteria, though this could also be related to the fact that trophic alterations are linked to hydrological alterations dropping the salinity, thus making the water less saline and consequently less suitable for Halobacteria. As for the restored lakes, the genera *Parvularcula* and *Phaeodactylibacter* were the characteristic taxa. In the lakes with hydrological alteration, the characteristic taxon with the highest relative abundance was the genus *Enhydrobacter*, while in the lakes experiencing both hydrological and trophic alteration, the characteristic taxa were the genera *Aquiflexum* and *Tetrasphaera*.

On the other hand, in the sediments (Figure 4B), the well‐preserved lakes had the family Carnobacteriaceae and the genus *Pseudooceanicola* as characteristic taxa. In the restored lakes, the phylum Bacteroidota was the most characteristic taxon, also showing a high relative abundance. In the hydrologically altered lakes, the class Desulfobacteria was the most characteristic and abundant taxon, although the cyanobacterial genus *Lyngbya*, despite its low relative abundance, also showed an important weight as a characteristic taxon. The lakes with hydrological and trophic alteration, however, did not show any characteristic taxon.

### 3.3 Methanogenic and Methanotrophic Taxa

**FIGURE 5:**
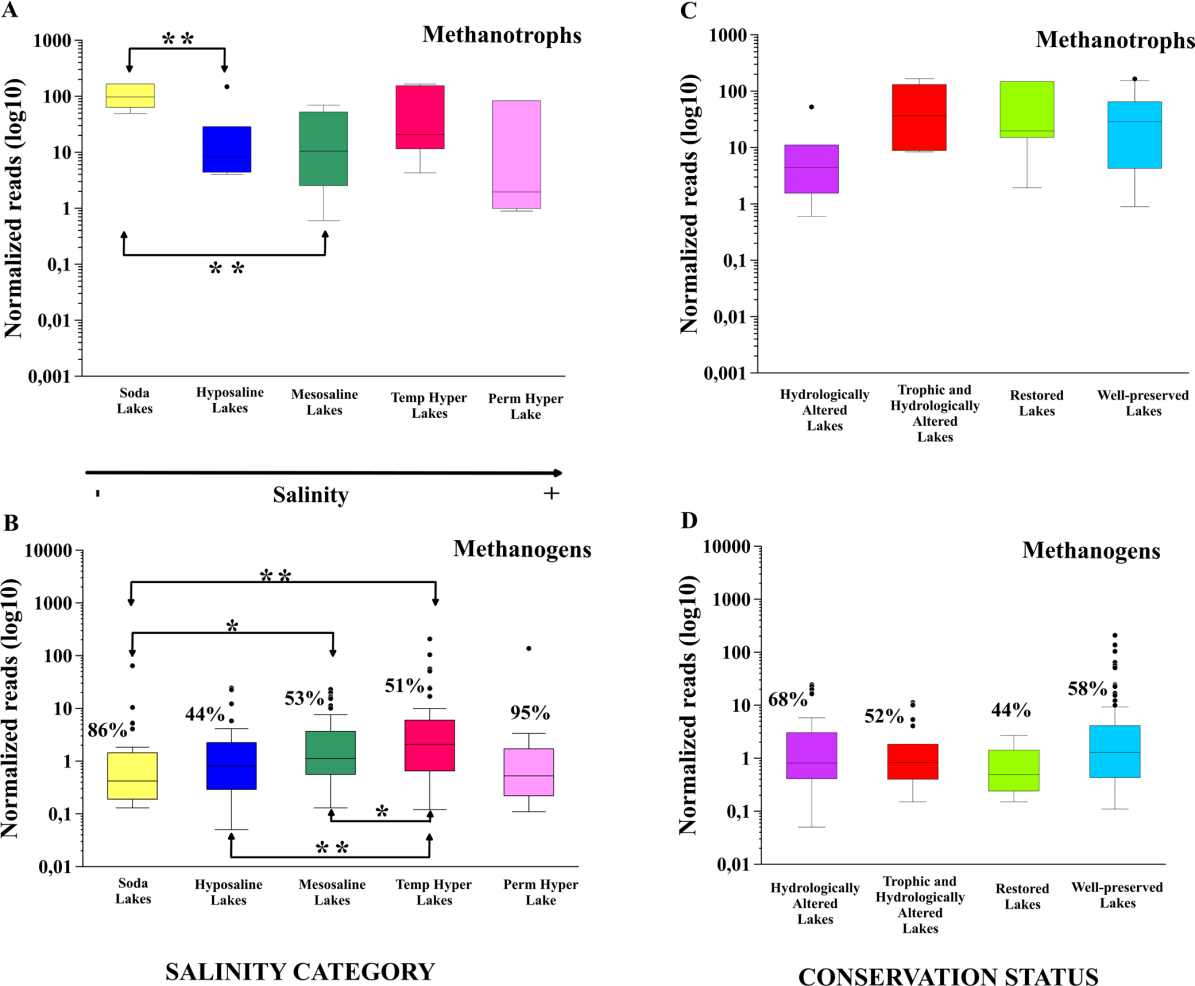
Box plots showing the distribution of the relative abundance (in logarithmic scale) of aerobic methanotrophic bacteria in water (A, C) and methanogenic archaea in sediment (B, D) across the different salinity categories and conservation statuses. The inner line of each box plot represents the median. The percentage of Methanomassiliicoccaceae reads with respect to the total methanogen reads in each salinity category and conservation status is indicated next to the respective box plots. Outliers were not taken into account in the statistical analyses. *p < 0.05, **p < 0.01.

The distribution of aerobic methanotrophic bacteria and methanogenic archaea showed different trends across salinity categories and conservation statuses (Figure 5).

Concerning methanotrophic bacteria, the most abundant family was Methylococcaceae. The highest relative abundance of methanotrophic bacteria in the water (Figure 5A), was found in the soda lakes, being significantly higher in these lakes compared to the hyposaline and mesosaline lakes, but no differences were observed with the rest of the more saline lakes. Furthermore, no significant differences were observed in the relative abundance of methanotrophic bacteria in the water between the different conservation statuses (Figure 5C), although the lakes with hydrological alteration showed a lower abundance than the rest of the lakes. Regarding methanogenic archaea in the sediments, their relative abundance tended to increase along the salinity gradient (Figure 5B), and the most abundant family in all the salinity categories and conservation statuses was the family Methanomassiliicococcaceae, which performs methylotrophic methanogenesis, while other minoritarian families were Methanobacteriaceae and Methanoregulaceae. The soda lakes presented a low abundance of methanogens and differed significantly from the mesosaline and temporary hypersaline lakes. Hyposaline lakes and mesosaline lakes presented a significantly lower abundance of methanogens compared to temporary hypersaline lakes. On the other hand, the relative abundance of sediment methanogens remained at similar levels regardless of the conservation status (Figure 5D), with no significant differences between them.

### 3.4 In Situ Carbon‐Related Metabolisms and Molecular Inference

**FIGURE 6:**
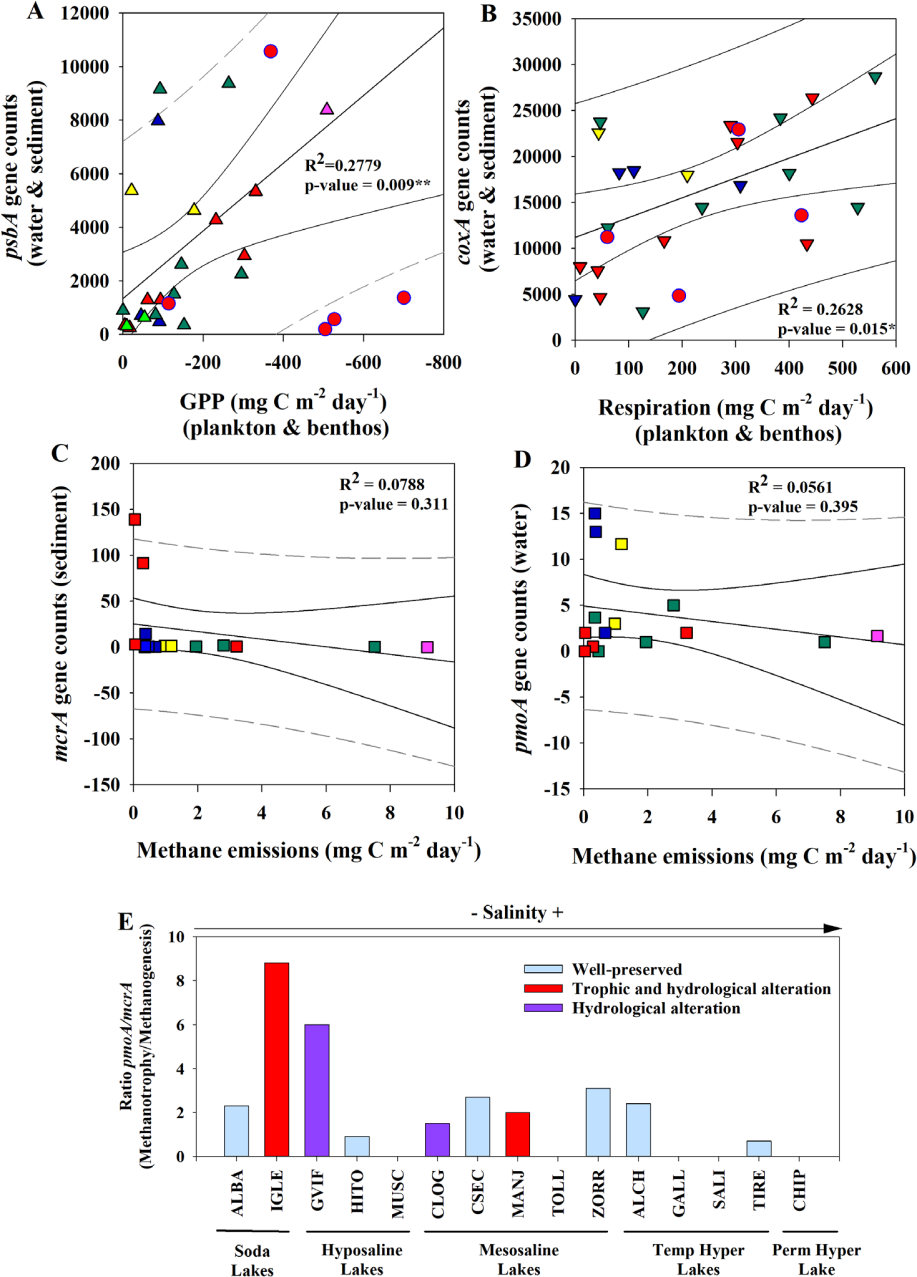
Linear regressions between lake averages of metabolic rates and gene counts of the respective marker genes obtained with PICRUSt2. The solid curved lines represent the 95% confidence intervals, and the dotted curved lines represent the prediction interval. Symbol color legend as in Figure 1. In A and B, values corresponding to altered lakes (●) were removed from the analysis. (A) Regression between GPP rates and *psbA* gene counts from water and sediment. (B) Regression between aerobic respiration rates and *coxA* gene counts from water and sediment. (C) Regression between methane emissions and gene counts of the *mcrA* gene from sediment. (D) Regression between methane emissions and gene counts of the *pmoA* gene from water. (E) Ratio between *pmoA* gene counts (potential methanotrophy) from water and *mcrA* gene counts (potential methanogenesis) from sediment. In (E), the absence of bars in certain lakes is due to the fact that in these lakes the gene counts of the *mcrA* gene were 0. *p < 0.05, **p < 0.01.

The relationship between the actual metabolic rates of the studied metabolisms and their gene counts inferred with PICRUSt2 is represented in Figure 6.

For gross primary production (GPP) and aerobic respiration, a significant positive regression was observed between the average actual rates and average gene counts of the respective marker genes (Figure 6A,B). However, the altered lakes were excluded from this regression as they did not show a direct relationship between measured rates and gene inference. Average methane emissions did not show any significant regression with average gene counts of methanogenesis marker genes in the sediment nor with methanotrophy marker genes in the water (Figure 6C,D).

Despite this, the ratio of gene counts *pmoA*/*mcrA* was higher in the altered lakes with low salinity, while in the hypersaline lakes this ratio was very low (Figure 6E), indicating that the potential aerobic methanotrophy was higher with respect to the potential methanogenesis in the less saline lakes, especially if they were altered. Regarding the different potential methanogenic pathways, few gene counts of the methylotrophic methanogenesis marker genes (*mtmB* and *mtbB*) were found, while the inference of the gene counts of the aceticlastic methanogenesis (*ackA*) and CO_2_ reduction methanogenesis (*mch*) marker genes gave high values in both water and sediment, and in the case of sediment did not show any significant relationship with methane emissions.

#### 3.4.1 Prokaryotic Co‐Occurrence Networks

The water and sediment networks are represented in Figures S1 and S2. The water network presented a clustering coefficient (reporting the complexity of the network) of 0.602 and a modularity (reporting the degree of compartmentalization of the network) of 0.789, showing a higher complexity and compartmentalization compared to the sediment network, which presented a clustering coefficient of 0.515 and a modularity of 0.633.

**FIGURE 7:**
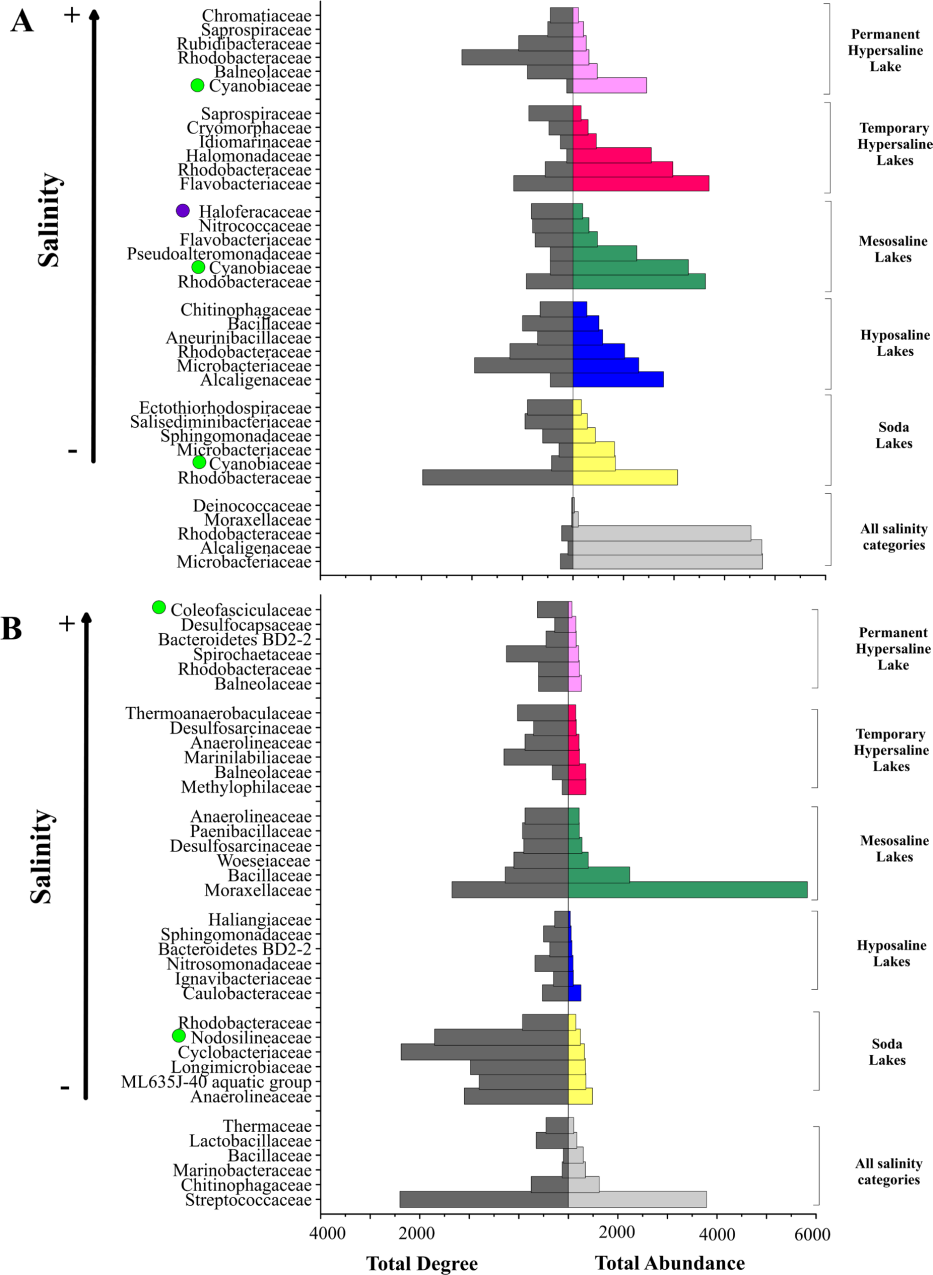
Total abundance and total degree of the six most abundant families in the specific networks of each salinity category for water (A) and sediment (B). Cyanobacterial families are highlighted with a green circle and archaeal families with a purple circle.

**FIGURE 8:**
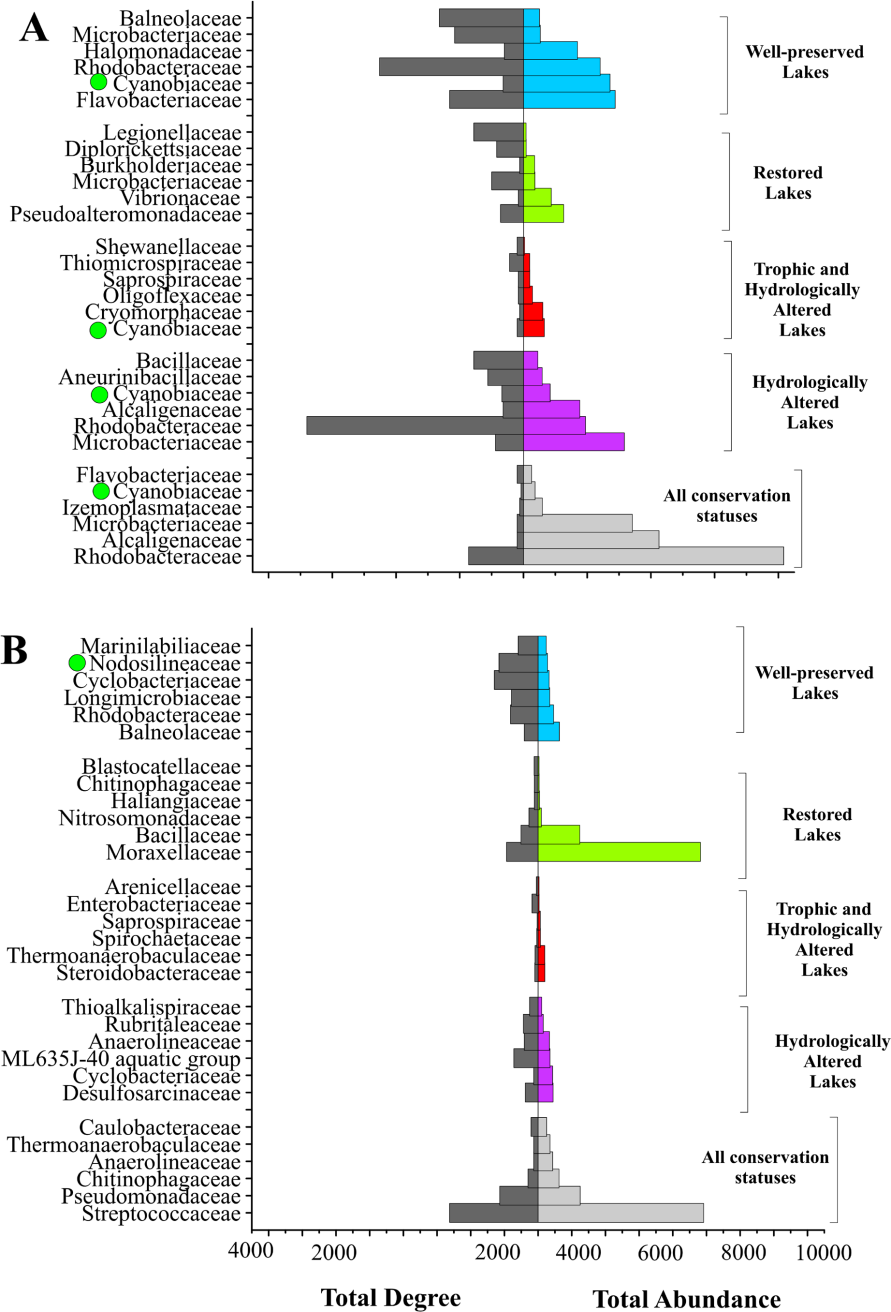
Total abundance and total degree of the 6 most abundant families in the specific networks of each conservation status for water (A) and sediment (B). Cyanobacterial families are highlighted with a green circle.

The total abundance and total degree of the six most abundant families in the specific networks of each salinity category and conservation status are respectively represented in Figures 7 and 8.

Regarding the different salinity categories, both water and sediment presented ZOTUs that were cosmopolitan and present in all salinity categories. These ZOTUs belonged to different families, with the families Microbacteriaceae, Alcaligenaceae, Rhodobacteraceae, and Streptococcaceae being the most abundant among them. In water (Figure 7A), cosmopolitan families had a high total abundance but a very low total degree, while in sediment (Figure 7B) the cosmopolitan family Streptococcaceae had both a high total abundance and total degree. In water (Figure 7A), the specific families of the different salinity categories generally showed either low total abundance but high total degree or high total abundance but low total degree. This pattern was exemplified by the families Cyanobiaceae and Haloferacaceae. The family Cyanobiaceae (phylum Cyanobacteria), which was present in almost all the water salinity categories, showed a very high total abundance but a very low total degree, while the family Haloferacaceae (Archaea), which was present in the mesosaline lakes, was the less abundant family but with the highest total degree. In comparison, in the sediment (Figure 7B) the total abundance of the specific families of the different salinity categories was lower than in water, but the total degree was at the same level as in water. However, the families Moraxellaceae and Bacillaceae had a very high total abundance in the mesosaline lakes. Moreover, in the sediment, the highest total degrees were found in the lowest saline lakes (soda lakes).

The patterns described for water and sediment were repeated in the different conservation statuses (Figure 8). The cosmopolitan families, whose ZOTUs are present in all the conservation statuses, had a very high total abundance but a very low total degree, except for the family Streptococcaceae in the sediment, which had a high total abundance and a high total degree. In the water samples (Figure 8A), in general, families with a high total abundance had a low total degree and vice versa, and the patterns described above for the family Cyanobiaceae were repeated, being one of the most abundant families in the different conservation statuses but showing a low total degree. As for the sediment (Figure 8B), when compared to the water, it was also observed that, generally, the most abundant families of each conservation status presented a lower total abundance in the sediment than in the water. In addition, in both the water and the sediment, the well‐preserved lakes had, in general, the highest total degrees.

With respect to the distribution of ZOTUs in the different sampling periods (Figure S3), in the sediment network the number of ZOTUs present in all the sampling periods (C) was very high compared to that of the water, and also the number of these ZOTUs was higher than the number of ZOTUs specific to any particular period, so the vast majority of sediment ZOTUs were not seasonal and were always present over time, while in the water most ZOTUs were seasonal and there were few ZOTUs present in all the sampling periods (C).

Regarding the topological role of the nodes that make up the network (Figure S4), in both the water and sediment networks, most nodes were classified as peripheral. In addition, some peripheral nodes showed very high abundances, while the abundances of other peripheral nodes were low. Nodes classified as module hubs and as connectors were only found in the sediment network. The module hub node was assigned to the Cyclobacteriaceae family, and the connector nodes were assigned to the sulfate‐reducing bacteria families Desulfococcaceae and Desulfatiglandaceae, as well as to the taxon Bacteroidetes BD2‐2. With regard to the genes related to carbon and sulfur metabolisms, both in the water and sediment, they were present in the peripheral nodes, and also in the connector nodes in the sediment, as some of them presented the *dsrB* gene. In the water, the most represented gene was *dsrB* (present in 18 nodes) followed by the *psbA* gene (present in 10 nodes). In the sediment, the most represented gene was *dsrB* (present in 43 nodes) followed by *psbA* (present in 7 nodes).

#### 3.4.2 Environmental Gradients and Structuration of the Prokaryotic Communities

**FIGURE 9:**
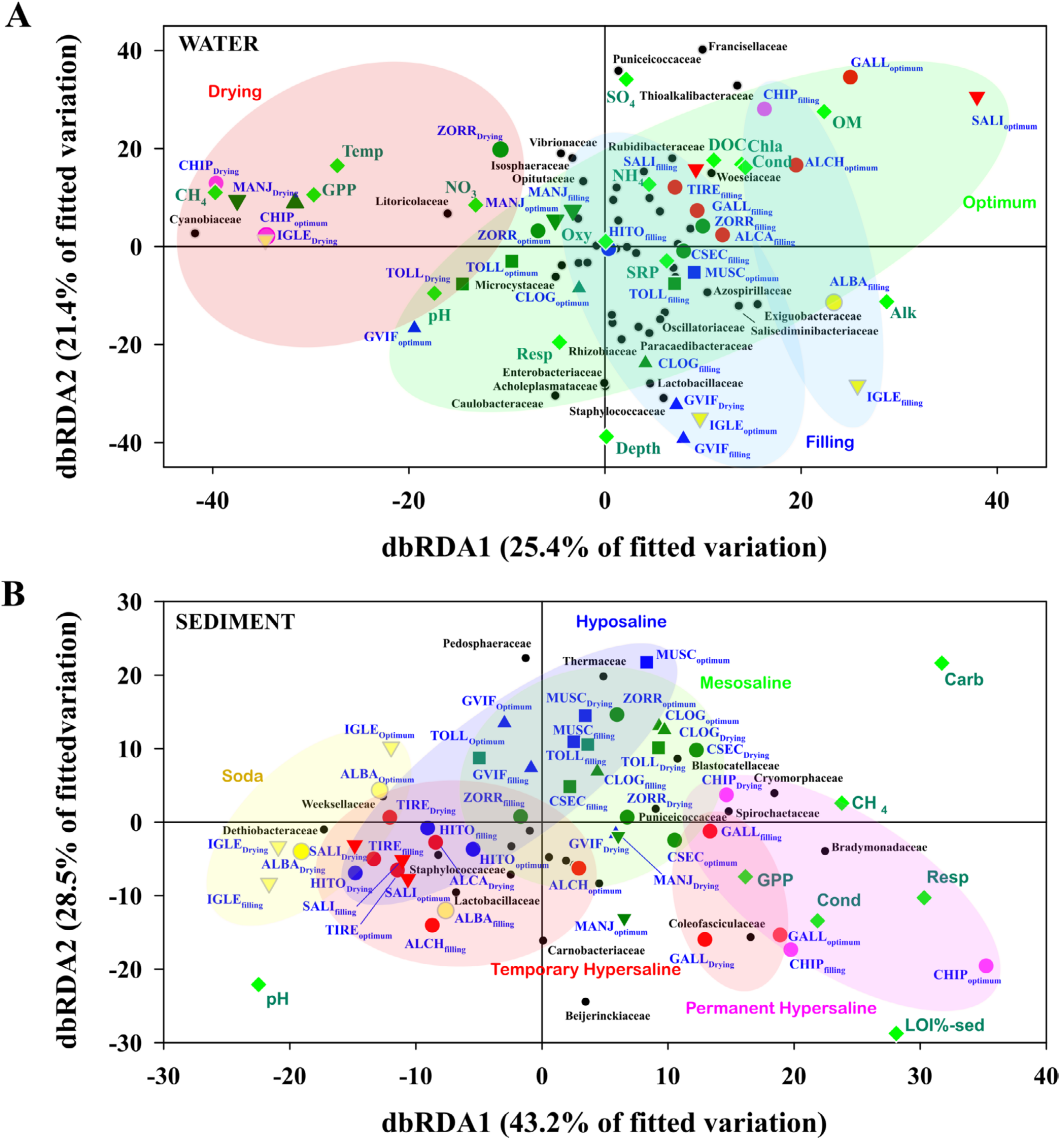
dbRDA showing the ordination of the water (A) and sediment (B) prokaryotic communities based on the environmental variables. The lake codes are described in Table 1. Symbol color legend as in Figure 1. In (A), the color of the ellipses indicates the sampling period: Blue (filling), green (ecological optimum) and red (drying). In (B), the color of the ellipses indicates the salinity category: Yellow (soda lakes), blue (hyposaline lakes), green (mesosaline lakes), red (temporary hypersaline lakes) and pink (permanent hypersaline lake). The shape of the symbols representing the lakes indicates their conservation status: Well‐preserved (●), restored (■), hydrologically altered (▲) or hydrologically and trophic altered (▼). Water environmental variables: Chla (chlorophyll‐*a*), Alk (alkalinity), OM (particulate organic matter), DOC (dissolved organic carbon), NH_4_ (ammonium), Oxy (oxygen), Temp (temperature), SRP (soluble reactive orthophosphate), Cond (conductivity), NO_3_ (nitrate), SO_4_ (sulfate), Depth (maximum depth), and pH. Sediment environmental variables: Cond (conductivity), Carb (carbonate), LOI%‐sed (sediment organic matter) and pH. Metabolic rates: GPP (gross primary production), Resp (aerobic respiration), and CH_4_ (methane emissions).

The classification of the inland saline lakes into different salinity categories and conservation statuses was reflected in the different environmental factors affecting the prokaryotic communities (Figure 9).

The water communities were determined by a combination of salinity category, conservation status, and seasonality (Figure 9A), which accounted for 27% of the total variation. The salinity category explained 13% of the total variation, while the alteration explained 8% and the season explained 5%. The interaction between these factors explained less than 1% of the total variation. Thus, the communities of the soda lakes were influenced by low conductivities and high alkalinity levels and showed a higher abundance of the families Exiguobacteraceae and Salisediminibacteriaceae. The communities of the hyposaline and mesosaline lakes were influenced by low to medium DOC values and showed a higher abundance of the families Opitutaceae, Microcystaceae, and Rhizobiaceae. The communities of the hypersaline lakes were associated with high concentrations of sulfate and organic matter, and the families Thioalkalibacteraceae and Francisellaceae were the most represented in these lakes. Furthermore, the combination of alteration and seasonality was shown to be a very important factor for the community structure of the aquatic communities of the altered lakes, as it separated the samples of warmer periods of these lakes, associated with high rates of gross primary production (GPP) and methane emissions and a high abundance of the Cyanobiaceae family, from their respective colder samples.

As for the sediment communities (Figure 9B), the prokaryotic communities were mainly affected by the salinity category to which the respective lakes belonged, followed by alteration and seasonality. These three factors explained 20% of the total variation. The salinity category explained 8% of the total variation, while the alteration explained 6% and the season explained 5%. The interaction between these factors explained less than 1% of the total variation. Thus, the communities of the soda lakes were influenced by low conductivities and high pH values, and the most abundant families were Weeksellaceae and Dethiobacteraceae. The communities of the hyposaline and mesosaline lakes were affected by high carbonate concentrations and showed intermediate GPP and aerobic respiration rates, along with high methane emission rates. The families Puniceicococcaceae, Thermaceae, and Blastocatellaceae were the most abundant in these lakes. The communities of the permanent hypersaline lake (Salada de Chiprana) and the temporary hypersaline lake (Laguna de Gallocanta) were influenced by high organic matter values and showed high rates of GPP and aerobic respiration. The most abundant families in their communities were Bradymonadaceae and Coleofasciculaceae. The communities of the rest of the temporary hypersaline lakes were scarcely affected by conductivity and formed a group together with the communities of the hyposaline lake Laguna de El Hito. These lakes showed very low methane emissions and also a high abundance of the family Staphylococcaceae in their communities.

## 4 Discussion

In this work, we studied the influence of salinity levels, conservation status, and seasonality on the structure and potential carbon‐related metabolisms of prokaryotic communities inhabiting the water and sediment of 15 Mediterranean inland saline shallow lakes. Each of these three lake groups, based on the above‐mentioned qualitative parameters, displayed specific prokaryotic communities and characteristic taxa.

In an overview of the prokaryotic communities of the studied inland saline lakes, the family Rhodobacteraceae and the phylum Cyanobacteria in the water and the family Streptococcaceae in the sediment stand out for their high relative abundances and wide distribution. In the water of these lakes, the family Rhodobacteraceae is cosmopolitan and is found along the entire salinity gradient. This family has a very wide distribution and is mainly found in marine environments (Simon et al. 2017), but has also been described in saline lakes (Zhong et al. 2015). In addition, its members possess great metabolic versatility (Pujalte et al. 2014; Simon et al. 2017), which could explain their ability to cope with the different conditions present in inland salt lakes. On the other hand, the phylum Cyanobacteria is abundant in the water of the studied lakes, especially during the warmer months in the lakes that are hydrologically and trophically altered. This type of alteration leads to a reduction in salinity, an increase in phosphorus concentrations, as well as an artificial elongation of the hydroperiod (Corrales‐González et al. 2019), with nutrient‐rich water in the warmer months of the year, when the lakes should be naturally dry, leading to a large growth of aquatic cyanobacterial populations. On the other hand, in the sediments, the family Streptococcaceae shows a high relative abundance in many of the temporary hyposaline and hypersaline lakes. These lakes are located in La Mancha Húmeda Biosphere Reserve, one of the largest wetland districts in the Iberian Peninsula (Corrales‐González et al. 2019) and support large waterfowl populations (Gonçalves et al. 2018). The Streptococcaceae likely come from bird feces and accumulate in the sediment of these lakes, thus smothering the effect of salinity on the community structure of their sediments. The importance of the effect of waterfowl on the sediment of endorheic saline lakes is highly underestimated, as only a few studies have shown that waterbird aggregations deeply affect the dynamics of microorganisms and nutrients in these lakes (Batanero et al. 2017).

On the other hand, the different salinity categories showed indicator taxa, as also described, though with lower lake types diversity, in another study in Spanish ephemeral saline lakes (Menéndez‐Serra et al. 2021). Nonetheless, the type of alteration of the lakes also affected their prokaryotic communities, leading to different indicator taxa between the conservation statuses. The ability of the indicator taxa to statistically discriminate a given salinity category or conservation status does not directly depend on their weight in the prokaryotic community, since, although some indicator taxa have a high relative abundance, most of them are minority organisms in the community. This pattern is well observed both in cyanobacteria and archaea. The cyanobacteria that act as characteristic aquatic taxa present a very uneven relative abundance, being majoritarian in the permanent hypersaline lake (Salada de Chiprana), where the genus *Synechococcus* represents almost 14% of the total reads, while in the lakes with hydrological alteration, cyanobacteria are not so abundant in the community. With respect to archaea, they generally display a low population weight, but they are key taxa for differentiating between salinity categories and conservation statuses, especially in the aquatic communities of the studied lakes. In fact, our work shows that archaea, specifically the class Halobacteria, are more represented in the aquatic communities of well‐preserved saline lakes, indicating that they could be a biomarker of good conservation status in inland saline ecosystems. In fact, this class of archaea consists of the organisms with the highest salt requirements and the highest resilience to high salinity in the archaea domain (Cui and Dyall‐Smith 2021). Since the alterations suffered by the studied inland saline lakes involve a reduction of their natural salinity due to artificial water input (Corrales‐González et al. 2019), it can be assumed that this will negatively affect this class of microorganisms that thrive in hypersaline waters, and that a reduction in the abundance of these organisms in any saline lake may indicate a hydrological alteration, and possibly also a trophic alteration of the lake, and therefore a degraded conservation status. On the other hand, for the sediments, both the mesosaline and the hydrologically and trophic altered lakes did not show characteristic taxa, which may indicate that, despite being classified within the same salinity category or conservation status, the prokaryotic communities of the different lakes in each of these two groups are very different from each other and the analysis of characteristic taxa does not find any taxa that are significantly more abundant in all the mesosaline nor in all the hydrologically and trophic altered lakes. In the case of the mesosaline lakes, this may be due to the large differences in salinity between the lakes in this group, with some of them having a salinity closer to that of the hyposaline lakes, and others having a salinity closer to that of the hypersaline lakes. This may result in mesosaline lakes with lower conductivities having a different prokaryotic community than mesosaline lakes with higher conductivities, so it is not possible to detect taxa that are characteristic of all the mesosaline lakes. In the case of the hydrologically and trophic altered lakes, their taxonomic profiles are so different that it is not possible to detect taxa common to both of them either.

Regarding carbon‐related metabolisms in the studied lakes, when comparing the metabolic rates measured in previous works (Camacho et al. 2017; Morant 2022) with the inferred abundances of their corresponding marker genes, it can be observed that gross primary production (GPP) and aerobic respiration show a significant correlation between their respective metabolic rates and marker genes, while methane‐related metabolisms, namely methanogenesis and aerobic methanotrophy, do not show a clear correlation. Regarding methanogenesis, this lack of correlation may be due to the lack of representation of genomes of the genus *Methanomassiliicococcus* in the genome database used by PICRUSt2 to infer gene abundances (database based on the IMG/M database, which collects microbial genomes), since there are only two representatives of this genus in the database. This is the only known genus of the family Methanomassiliicococcaceae, which is in turn the major family of methanogens in the sediment of the studied saline lakes. The lack of more genomes corresponding to undiscovered species of this family, very abundant in the saline lakes, may be related to the generally low number of inferred copies of the marker gene for methanogenesis (*mcrA*), and therefore to the lack of correlation obtained between this gene and methane emissions. Furthermore, this would also explain the low copy number of the *mtmB* and *mtbB* genes, marker genes for methylotrophic methanogenesis, which the only described and purely cultured species of the family Methanomassiliicococcaceae, *Methanomassiliicococcus luminyensis*, is capable of (Dridi et al. 2012; Kröninger et al. 2017). Methylotrophic methanogenesis has been described as being predominant in saline environments (Zhou et al. 2022), since its substrates are methylamines, which are abundant in saline environments allowing the activity of methylotrophic methanogens as they do not enter into competition with sulfate‐reducing bacteria, which do not use methylamines (Sorokin and McGenety 2019). Moreover, the high inferred copy number of the marker genes for aceticlastic methanogenesis (*ackA*) and methanogenesis by CO_2_ reduction (*mch*) in water, an aerobic environment where this type of methanogenesis cannot take place, indicates that the use of the *mcrA*, which is widely supported by the literature, is more indicated for the study of methanogenesis, as the *ackA* and *mch* genes are likely to have such an inferred abundance in water because they are not as specific as the *mcrA* gene and may be involved in metabolisms other than methanogenesis. As for the potential aerobic methanotrophy, although when considering all the lakes there is no correlation with methane emissions, in the altered lakes with lower salinity a higher ratio of *pmoA*/*mcrA* gene abundances is observed, indicating that in these lakes there may be a higher activity of methanotrophic bacteria due to an increase in methanogenesis as a consequence of this alteration. In fact, previous work in these same lakes has shown that the anthropogenic alteration of the lakes is related to higher methane emissions while elevated salinity reduces them (Camacho et al. 2017; Morant 2022). The relationship of methanogenesis with salinity is complex, and it could be the case that in the studied saline lakes the production of methylamines, the main substrate of methylotrophic methanogenesis, is low, and therefore that methane production could depend mainly on methanogenesis from acetate or CO_2_, metabolic pathways that are activated in the less saline lakes, which naturally show a low salinity and therefore low sulfate levels. This may also happen in the altered lakes, which present a lower salinity due to the artificial inflows of freshwater, which implies lower competition between methanogens that use these two methanogenic pathways and sulfate‐reducing bacteria, since reducing salinity also reduces the sulfate that these bacteria need. Furthermore, in lakes with hydrological and trophic alteration, this reduction in salinity due to freshwater inflows can be accompanied by an increase in nutrients and organic substrates that can be used by aceticlastic or CO_2_‐reducing methanogens, which therefore increase their activity.

Apart from its effects on the prokaryotic community structure and carbon‐related metabolisms, salinity is also a main determining factor for the organization of prokaryotic communities, as observed in co‐occurrence networks (Wang et al. 2021; Yang et al. 2021). In the communities of the studied saline lakes, all different salinity categories and different conservation statuses present cosmopolitan ZOTUs, which show high abundance, but at the same time very low degree (number of connections) with the rest of the members of the networks, especially in water communities. This can be interpreted as meaning that only a few ZOTUs show a high adaptability and can thrive in all the salinity categories and conservation statuses. At the same time, the strong differences in the environmental characteristics between the different salinities and conservation statuses lead to the co‐occurrence of prokaryotes that are very specific to each salinity category and conservation status but that are not present in the other ones. On the other hand, and in opposition to the findings of Yang et al. (2021), focusing on the salinity level (category), in sediment networks the highest degrees are observed in the less saline lakes, whereas regarding the conservation status, both in water and sediment the well‐preserved lakes showed the highest degrees. On one hand, this reduction in the number of connections between community members as salinity increases may reflect simpler communities in the most saline lakes. But, on the other hand, human‐induced alterations exert an important effect on the topology of co‐occurrence networks (Hu et al. 2017) and tend to simplify them (Karimi et al. 2017). Since the highest number of connections between members of saline lakes communities has also been observed in the well‐preserved lakes, it can be assumed that anthropogenic pressures have led to a simplification of interactions between microorganisms in the prokaryotic communities of altered inland saline lakes. Recently, the idea of using microbial networks as markers of ecosystem quality and functioning has been developed (Karimi et al. 2017), but a thorough understanding of microbial networks in the best‐preserved environments is needed before developing indices or markers of ecological status based on them, since, for example, it might be thought that fewer connections between community members in the most saline lakes is a consequence of human pressure, whereas, in fact, this is a natural feature.

Furthermore, the different taxonomic groups into which the members of the co‐occurrence networks are grouped may have a different pattern of connections. In the case of the saline lakes, cyanobacteria and archaea follow a disparate pattern. In this way, cyanobacteria have a very high abundance in the network, but at the same time, they have a very little degree, resulting in very abundant cyanobacterial populations, but poorly connected to the rest of the community, as has been observed in some eutrophic lakes (Zhao et al. 2016). Cyanobacteria, being photosynthetic organisms with a very specific ecological niche, co‐occur mainly with other Cyanobacteria and with the microbiota associated with them. Regarding archaea, they have a low abundance in the co‐occurrence network, but at the same time, they have a high degree. This fact underlines the consideration of archaea as key members of microbial communities (Moissl‐Eichinger et al. 2018) and leads to the conclusion that archaea in inland saline lakes may be considered keystone taxa, which are those whose disappearance would be expected to cause very large deleterious effects on the community (Berry and Widder 2014; Goberna and Verdú 2022). Due to the large number of connections that archaea present within co‐occurrence networks, any factor affecting them could trigger large changes in the relationships between community members that would be shown by the co‐occurrence networks.

When considering the interaction between environmental variables, the rates of carbon‐related metabolisms, and the distribution and abundance of prokaryotic communities, differential patterns have been observed between water and sediment. The structuring of water communities is the result of the interaction between salinity, anthropogenic alteration, and seasonality. Seasonality deeply influences metabolic rates, with samples from warmer periods in altered lakes exhibiting higher rates of gross primary production. This is consistent with other works for different types of organisms, where salinity and temperature are shown to be very important factors in, for instance, the phytoplankton community structure (Lawrenz et al. 2013; López‐Flores et al. 2014; Nche‐Fambo et al. 2015), and underlines the fact that any alteration of the hydroperiod, or artificial input of nutrients, strongly affects the structure and carbon metabolisms of prokaryotic communities in inland saline lakes. In fact, the anthropogenic alteration of the lakes deeply influences their carbon cycle and its metabolic rates (Camacho et al. 2017; Morant 2022; Camacho‐Santamans et al. 2024; Morant et al. 2024). In altered lakes, phytoplankton rates of gross primary production are higher, initially leading to an increased capacity of carbon sequestration. Nonetheless, their carbon‐emitting metabolisms, especially methanogenesis, also increased. A holistic view of the ecosystem is therefore necessary to assess the effect of anthropogenic alterations on the carbon cycle in such saline lakes. Thus, in the altered lakes, the longer hydroperiods due to the artificial water inputs lead to the presence of water during summer, when these lakes should be naturally dry. At the same time, the increased nutrient availability in the altered lakes together with the higher temperatures present during summer and the lower salinity trigger a higher methane production that counteracts the metabolisms linked to carbon sequestration. This combination of factors results in a higher activity of C‐GHG emitting metabolisms and a lower climate change mitigation capacity in altered lakes, while well‐preserved lakes show higher carbon retention and climate change mitigation capacity (Morant et al. 2024).

In addition, the high seasonality in water indicates that aquatic microbial communities have little temporal stability, probably because seasonal changes in environmental variables in water are more pronounced than in sediment, leading to large seasonal differences in aquatic prokaryotic communities. In fact, the water co‐occurrence network was more complex and compartmentalized than the sediment network, probably because of the seasonal changes in the prokaryotic communities, as most nodes of the water network were present only in specific seasons. Also, these seasonal changes may be affected by the anthropic alterations, leading, for example, to a higher abundance of Cyanobacteria during the warmer periods in altered lakes, which are linked to increased gross primary production rates and therefore to alterations in the carbon cycle in the altered lakes. In contrast, sediment communities are more stable over time and are more influenced by salinity categories than by the type of alteration or seasonality. Nevertheless, the input of exogenous microorganisms like those abundant in waterfowl drops in temporary hypersaline lakes strongly modifies the effect of salinity and should be considered a factor that could be of great importance in structuring sediment prokaryotic communities in small inland saline lakes with large waterfowl populations.

## 5 Conclusion

The results of our work show that the prokaryotic communities of the different salinity categories and conservation statuses into which the inland saline lakes are divided follow general patterns in their structuring and potential carbon metabolisms. Further than cosmopolitan taxa, each salinity category and conservation status shows characteristic microbial taxa. Potential gross primary production and respiration are significantly correlated with the respective measured metabolic rates, whereas the relationship between methane emissions and potential methane metabolisms is not so clear, probably because of the interaction of multiple factors. Furthermore, anthropogenic alteration and seasonality have been shown to have a significant impact on both the prokaryotic communities and rates of GPP and methane emissions from the studied saline lakes. The complexity of the interactions between these factors underlines the importance of combining molecular studies with in situ measurements of the rates of the main carbon‐related metabolisms to obtain a good overview of the factors controlling carbon fluxes in these types of ecosystems. This knowledge could improve the management of inland saline shallow lakes in the current climate change scenario, enhancing their carbon sequestration capacity while maintaining the compositional and functional biodiversity of these types of wetlands.

## Author Contributions

**Javier Miralles‐Lorenzo:** conceptualization (equal), data curation (equal), formal analysis (equal), investigation (equal), methodology (equal), writing – original draft (lead), writing – review and editing (equal). **Antonio Picazo:** conceptualization (equal), data curation (equal), formal analysis (equal), investigation (equal), methodology (equal), software (equal), supervision (equal), validation (equal), writing – review and editing (equal). **Carlos Rochera:** data curation (equal), investigation (equal), methodology (equal), supervision (equal), validation (equal). **Daniel Morant:** investigation (equal), methodology (equal). **Emilio O. Casamayor:** methodology (equal), software (equal), supervision (equal), validation (equal). **Mateu Menéndez‐Serra:** methodology (equal), software (equal), supervision (equal), validation (equal). **Antonio Camacho:** conceptualization (equal), funding acquisition (lead), investigation (equal), methodology (equal), project administration (lead), supervision (equal), validation (equal), writing – original draft (supporting), writing – review and editing (equal).

## Conflicts of Interest

The authors declare no conflicts of interest.

## Supporting information


Appendix S1.

## Data Availability

Raw Illumina 16S rRNA gene metabarcoding data have been deposited in the NCBI Sequence Read Archive under BioProject ID PRJNA819854.
