# Supplementary material for: Environmental Gradients and Conservation Status Determine the Structure and Carbon‐Related Metabolic Potential of the Prokaryotic Communities of Mediterranean Inland Saline Shallow Lakes
Source: Ecol Evol. 2025 May 26;15(5):e71286. doi: 10.1002/ece3.71286 (PMC12104873; doi:10.1002/ece3.71286)
Supplement: Supplementary file 1 — Appendix S1. [file ECE3-15-e71286-s001.docx]

**Supplementary Table 1**. Average values and standard deviation (SD) of the main environmental variables of the water of the studied lakes. These data correspond to the 2016-17 and 2017-18 hydrological cycles and have been used to determine the salinity categories to which the lakes belong and the alterations they suffer. Lake codes as in Table 1. *: hydrological alteration. **: hydrological and trophic alteration. Cond: Conductivity. Chl-*a*: Chlorophyll-*a*. TSS: Total Suspended Solids. OM: Particulate Organic Matter. DOC: Dissolved Organic Carbon. Alk: Alkalinity. SRP: Soluble Reactive Orthophosphate. NO_3_^-^. Nitrate. NH_4_^+^: ammonium. Depth: depth at the deepest point of the lake.

| **Lake** | **Cond (mS·cm^-1^)** | **SD** | **Chl-*a* (mg·m^-3^)** | **SD** | **pH** | **SD** | **TSS (mg·L^-1^)** | **SD** | **OM (mg·L^-1^)** | **SD** | **DOC**  **(mg C·L^-1^)** | **SD** | **Alk (meq·L^-1^)** | **SD** | **SRP (µM)** | **SD** | **NO_3_^-^(µM)** | **SD** | **NH_4_^+^**  **(µM)** | **SD** | **Depth**  **(cm)** | **SD** |
| --- | --- | --- | --- | --- | --- | --- | --- | --- | --- | --- | --- | --- | --- | --- | --- | --- | --- | --- | --- | --- | --- | --- |
| ALBA | 3.7 | 1.5 | 0.6 | 0.8 | 10.0 | 0.5 | 12.3 | 14.0 | 6.0 | 7.4 | 34.2 | 18.4 | 26.4 | 16.6 | 2.1 | 0.6 | 47.8 | 12.8 | 8.7 | 7.3 | 21 | 15 |
| IGLE** | 5.8 | 4.9 | 16.3 | 26.0 | 9.2 | 0.6 | 53.8 | 71.4 | 20.0 | 26.5 | 59.4 | 33.9 | 35.6 | 12.0 | 3.2 | 2.2 | 138.1 | 102.9 | 16.0 | 11.6 | 44 | 5 |
| GVIF* | 9.9 | 2.5 | 2.5 | 2.8 | 8.4 | 0.4 | 12.9 | 7.3 | 5.4 | 3.4 | 19.8 | 7.5 | 3.0 | 0.7 | 0.6 | 1.0 | 52.3 | 31.2 | 12.7 | 10.1 | 78 | 36 |
| HITO | 8.6 | 4.4 | 0.9 | 0.1 | 8.1 | 0.1 | 60.9 | 42.9 | 26.4 | 20.3 | 13.0 | 8.3 | 1.4 | 0.2 | 0.2 | 0.1 | 14.9 | 11.5 | 8.7 | 5.9 | 7 | 2 |
| MUSC | 4.0 | 3.0 | 0.7 | 0.9 | 7.5 | 0.1 | 8.9 | 5.4 | 4.8 | 0.1 | 24.0 | 0.8 | 6.2 | 0.1 | 1.0 | 0.5 | 29.4 | 16.3 | 9.5 | 0.1 | 14 | 8 |
| CLOG* | 35.7 | 20.6 | 1.8 | 2.1 | 8.1 | 0.3 | 49.9 | 57.3 | 13.4 | 13.9 | 64.8 | 66.5 | 4.8 | 2.0 | 0.3 | 0.3 | 184.3 | 151.3 | 19.4 | 12.0 | 38 | 35 |
| CSEC | 38.7 | 29.2 | 9.8 | 16.7 | 8.9 | 0.1 | 76.7 | 60.4 | 15.3 | 10.6 | 21.7 | 24.9 | 6.3 | 2.0 | 1.0 | 1.5 | 88.4 | 53.1 | 96.3 | 121.1 | 15 | 7 |
| MANJ** | 35.3 | 32.3 | 24.1 | 23.8 | 8.5 | 0.4 | 226.5 | 316.7 | 93.7 | 123.3 | 71.7 | 58.1 | 10.9 | 7.9 | 2.7 | 4.2 | 219.1 | 245.0 | 24.6 | 22.4 | 11 | 10 |
| TOLL | 29.0 | 23.0 | 0.3 | 0.2 | 8.4 | 0.7 | 22.9 | 12.4 | 4.2 | 3.1 | 17.0 | 6.8 | 1.7 | 0.8 | 0.2 | 0.1 | 54.7 | 32.5 | 10.0 | 5.8 | 35 | 12 |
| ZORR | 35.2 | 51.8 | 3.8 | 4.5 | 8.1 | 0.8 | 172.8 | 173.5 | 31.4 | 31.3 | 63.7 | 85.5 | 4.8 | 4.9 | 0.5 | 0.5 | 183.1 | 257.7 | 17.0 | 22.5 | 15 | 5 |
| ALCH | 53.0 | 53.3 | 5.8 | 3.8 | 8.1 | 0.5 | 169.7 | 113.2 | 102.0 | 132.7 | 34.8 | 38.6 | 4.0 | 1.5 | 0.3 | 0.2 | 84.2 | 49.2 | 35.2 | 23.2 | 8 | 15 |
| GALL | 123.8 | 155.4 | 14.8 | 14.1 | 8.0 | 1.1 | 629.2 | 1079.6 | 202.0 | 343.9 | 158.4 | 159.7 | 8.1 | 5.2 | 0.6 | 0.6 | 499.4 | 444.5 | 105.6 | 123.1 | 5 | 2 |
| SALI** | 90.9 | 75.3 | 40.1 | 28.9 | 8.4 | 0.4 | 638.5 | 686.1 | 190.6 | 184.8 | 59.3 | 21.1 | 7.6 | 4.3 | 4.4 | 10.2 | 107.6 | 58.9 | 43.1 | 60.9 | 3 | 2 |
| TIRE | 93.3 | 99.2 | 6.9 | 4.0 | 8.3 | 0.3 | 1199.8 | 2601.0 | 298.1 | 619.2 | 47.4 | 52.2 | 9.2 | 11.5 | 4.1 | 5.8 | 65.3 | 42.9 | 25.4 | 20.3 | 8 | 4 |
| CHIP | 87.0 | 50.7 | 3.3 | 3.3 | 8.3 | 0.3 | 197.8 | 355.4 | 61.4 | 110.7 | 99.9 | 34.5 | 17.1 | 7.4 | 0.6 | 0.5 | 440.0 | 175.8 | 83.3 | 48.1 | 40 | 9 |


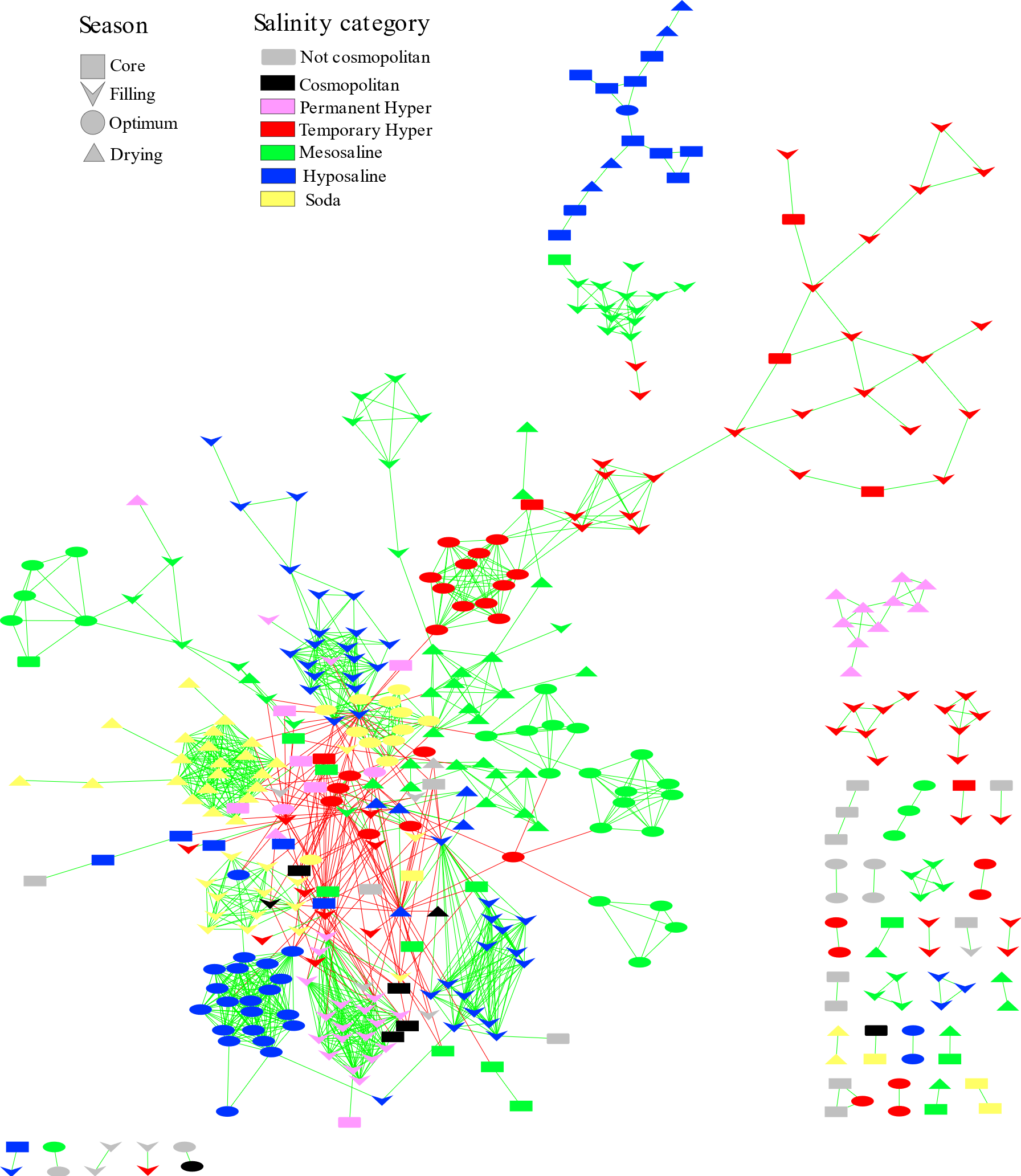


**Supplementary Figure 1.** Co-occurrence network of the water prokaryotic communities of the studied lakes. The results of the topological analysis of the network are reflected in Figures 7 and 8. ZOTUs are represented by nodes, and their relationships by edges. Green edges indicate a co-occurrence relationship and red edges a co-exclusion relationship. Node shape indicates the different levels of factor season, and node color indicates the different levels of factor salinity category. Each ZOTU was assigned a level for the factors season and salinity category if its abundance in any of these levels was greater than 70% of its total reads. The ZOTUs that did not reach a minimum abundance of 70% were divided into two classes. On the one hand, if within the factor salinity category, a ZOTU had reads at all levels of that factor, it was considered cosmopolitan. If the same was true for the season factor, with reads in all seasons, it was considered to be present throughout the year (Core). On the other hand, if a ZOTU had no reads at any of the levels of factor salinity category, it was considered not cosmopolitan.


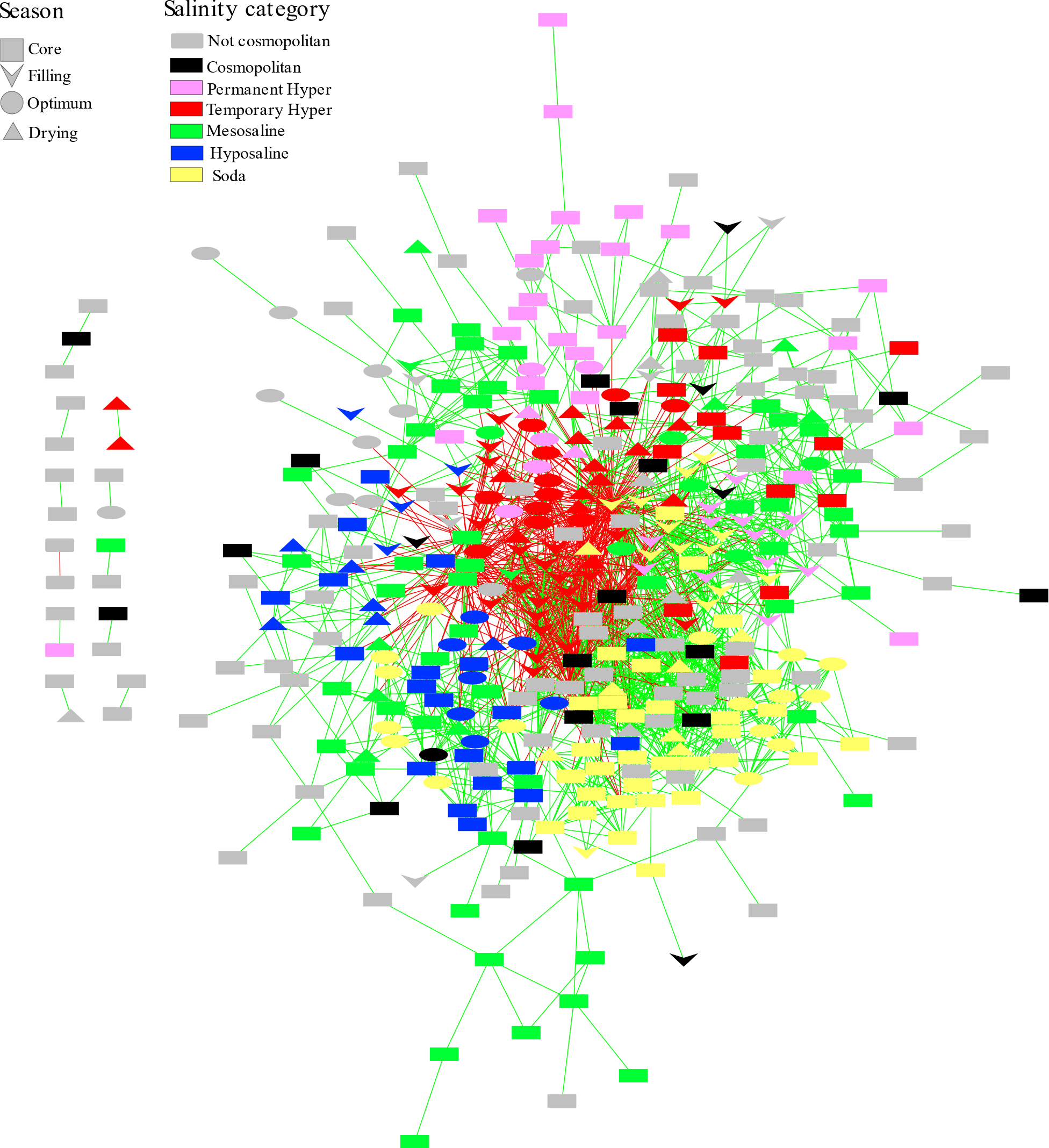


**Supplementary Figure 2.** Co-occurrence network of the sediment prokaryotic communities of the studied lakes. The results of the topological analysis of the network are reflected in Figures 7 and 8. ZOTUs are represented by nodes, and their relationships by edges. Green edges indicate a co-occurrence relationship and red edges a co-exclusion relationship. Node shape indicates the different levels of factor season, and node color indicates the different levels of factor salinity category. Each ZOTU was assigned a level for the factors season and salinity category if its abundance in any of these levels was greater than 70% of its total reads. The ZOTUs that did not reach a minimum abundance of 70% were divided into two classes. On the one hand, if within the factor salinity category, a ZOTU had reads at all levels of that factor, it was considered cosmopolitan. If the same was true for the season factor, with reads in all seasons, it was considered to be present throughout the year (Core). On the other hand, if a ZOTU had no reads at any of the levels of factor salinity category, it was considered not cosmopolitan.

**Supplementary Figure 3.** Distribution of the number of ZOTUs present throughout the year (core ZOTUS, indicated with a C) and of the ZOTUs specific to each sampling period in the water and sediment co-occurrence networks.

**Supplementary Figure 4.** Z_i_-P_i_ plot showing, for water and for sediment, the distribution of nodes forming the co-occurrence networks based on their topological roles. The size of the symbol representing the nodes is proportional to their abundance in the network, and the color of the symbol indicates which of the following genes is present in the ZOTU the node represents: yellow (*dsrB*) and green (*psbA*). Z_i_: within-module connectivity. P_i_: among-module connectivity.
